# A WD40-repeat protein unique to malaria parasites associates with adhesion protein complexes and is crucial for blood stage progeny

**DOI:** 10.1186/s12936-015-0967-x

**Published:** 2015-11-04

**Authors:** Andreas von Bohl, Andrea Kuehn, Nina Simon, Vanesa Nkwouano Ngongang, Marc Spehr, Stefan Baumeister, Jude M. Przyborski, Rainer Fischer, Gabriele Pradel

**Affiliations:** Division of Cellular and Applied Infection Biology, Institute for Biology II, RWTH Aachen University, Worringerweg 1, 52074 Aachen, Germany; Institute of Molecular Biotechnology, RWTH Aachen University, Worringerweg 1, 52074 Aachen, Germany; Research Center for Infectious Diseases, University of Würzburg, Josef-Schneider-Straße 2/D15, 97080 Würzburg, Germany; Department of Chemosensation, Institute for Biology II, RWTH Aachen University, 52074 Aachen, Germany; Parasitology Section, Faculty of Biology, Philipps University Marburg, Karl-von-Frisch-Straße 8, 35043 Marburg, Germany

**Keywords:** Malaria, *Plasmodium falciparum*, Merozoite, Microneme, Gametocyte, WD40, *Pf*AMA1, *Pf*s230, *Pf*CCp protein

## Abstract

**Background:**

During development in human erythrocytes, *Plasmodium falciparum* parasites display a remarkable number of adhesive proteins on their plasma membrane. In the invasive merozoites, these include members of the *Pf*MSP1 and *Pf*AMA1/RON complexes, which facilitate contact between merozoites and red blood cells. In gametocytes, sexual precursor cells mediating parasite transmission to the mosquito vector, plasma membrane-associated proteins primarily belong to the *Pf*CCp and 6-cys families with roles in fertilization. This study describes a newly identified WD40-repeat protein unique to *Plasmodium* species that associates with adhesion protein complexes of both merozoites and gametocytes.

**Methods:**

The WD40-repeat protein-like protein *Pf*WLP1 was identified via co-immunoprecipitation assays followed by mass spectrometry and characterized using biochemical and immunohistochemistry methods. Reverse genetics were employed for functional analysis.

**Results:**

*Pf*WLP1 is expressed both in schizonts and gametocytes. In mature schizonts, the protein localizes underneath the merozoite micronemes and interacts with *Pf*AMA1, while in gametocytes *Pf*WLP1 primarily accumulates underneath the plasma membrane and associates with *Pf*CCp1 and *Pf*s230. Reverse genetics failed to disrupt the *pfwlp1* gene, while haemagglutinin-tagging was feasible, suggesting a crucial function for *Pf*WLP1 during blood stage replication.

**Conclusions:**

This is the first report on a plasmodial WD40-repeat protein associating with cell adhesion proteins. Since WD40 domains are known to mediate protein–protein contact by serving as a rigid scaffold for protein interactions, the presented data suggest that *Pf*WLP1 supports the stability of adhesion protein complexes of the plasmodial blood stages.

**Electronic supplementary material:**

The online version of this article (doi:10.1186/s12936-015-0967-x) contains supplementary material, which is available to authorized users.

## Background

Protein complexes are formed by two or more non-covalently bound proteins mutually supportive in distinct cell functions. Protein complexes are crucial for most cell biological processes and among others function in establishing cell–cell contacts. Thus such complexes are of importance for interactions between pathogens and between pathogens and their host cells.

For intracellular pathogens, such as malaria parasites, recognition, adhesion and invasion of host cells are essential steps during infection and are often mediated by protein–protein interactions. A variety of protein complexes have previously been described for the human malaria parasite *Plasmodium falciparum*, like the actin-myosin motor complex enabling gliding motility of the invasive stages. Also, most of the adhesive proteins found on the surface of the invasive merozoites involved in binding to the red blood cell (RBC) prior to infection are known to assemble as complexes, e.g., the merozoite surface protein 1 (MSP1) complex or the apical membrane antigen 1 (AMA1)/Rhoptry neck (RON)-complex (reviewed in [1–3]).

The large MSP complex consists mainly of the GPI-anchored surface protein MSP1, the peripheral membrane proteins MSP6 and MSP7 and the recently found MSP-Duffy-binding like proteins MSPDBL1 and MSPDBL2 ([4], reviewed in [2, 3]). Although the exact mechanistic function of the MSP1 complex is not fully understood, it is suggested that the MSP complex is responsible for the initial interaction of the merozoite with the RBC. The AMA1/RON complex, on the other hand, is crucial for the formation of the tight junction during merozoite invasion. It was shown that AMA1, a transmembrane protein of the micronemal membrane, upon merozoite attachment to the RBC relocates to the plasma membrane and then interacts with RON proteins that have been secreted and inserted into the RBC membrane. Here, RON2 functions as an anchor on the RBC membrane, interacting with the parasite transmembrane protein AMA1 and thereby forming the tight junction ([5, 6], reviewed in [7]).

Protein complexes can also be found in the non-invasive, amotile gametocyte stages of *P. falciparum*. Gametocytes are dormant sexual precursor cells of the malaria pathogen, which differentiate in human RBCs and once taken up during the blood meal, become activated and transform into male and female gametes to initiate sexual reproduction (reviewed in [8]). A remarkable feature of gametocytes is the expression of numerous adhesive proteins, which are associated with the plasma membrane within the parasitophorous vacuole. These include the six-cysteine (6-cys) proteins *Pf*s230 and *Pf*s48/45 and the six *Limulus* coagulation factor C-like (LCCL)-domain containing *Pf*CCp proteins (reviewed in [9]). Noteworthy, the *Pf*CCp proteins assemble to protein complexes associated with the gametocyte plasma membrane via an interaction with *Pf*s230, which itself is bound to the GPI-anchored *Pf*s48/45 ([10–13], reviewed in [8]). Recent findings demonstrated a rearrangement of this protein complex during gametogenesis resulting into an increased interaction of the *Pf*CCp proteins to *Pf*s230 as well as to the GPI-anchored protein *Pf*s25 of macrogametes [14].

In an attempt to identify further interaction partners of the *Pf*CCp-based protein complex, in this study co-immunoprecipitation assays were employed and identified a WD40-repeat protein unique to the *Plasmodium* species that associates with selected adhesion protein complexes of merozoites and gametocytes. Since WD40-repeat domains are known to facilitate protein–protein contact, the data point to the WD40-repeat protein playing a role in promoting stability of the cell adhesion protein complexes in these stages.

## Methods

### Gene identifiers

The following gene identifiers are assigned to the proteins investigated in this study: *Pf*AMA1 [PlasmoDB: PF3D7_1133400]; *Pf*CCp1 [PlasmoDB: PF3D7_1475500]; *Pf*CCp2 [PlasmoDB: PF3D7_1455800]; *Pf*CCp4 [PlasmoDB: PF3D7_0903800]; *Pf*s48/45 [PlasmoDB: PF3D7_1346700]; *Pf*s230 [PlasmoDB: PF3D7_0209000]; *Pf*GAP45 [PlasmoDB: PF3D7_1222700]; *Pf*GAP50 [PlasmoDB: PF3D7_0918000]; *Pf*α-tubulin I [PlasmoDB: PF3D7_0903700]; *Pf*EXP1 [PlasmoDB: PF3D7_1121600]; *Pf*MSP1 [PlasmoDB: PF3D7_0930300]; *Pf*39 [PlasmoDB: PF3D7_1108600]; *Pf*WLP1 [PlasmoDB: PF3D7_1443400].

### Antibodies

The following antibodies were used in this study: mouse polyclonal antisera against *Pf*CCp1rp1, *Pf*CCp4rp1, and *Pf*39rp1 [12–14]; *Pf*s230 region C [15], *Pf*s48/45 (ATCC) and against the MBP-tag (Sigma-Aldrich); rabbit polyclonal antisera against *Pf*MSP1, *Pf*s230 region C, *Pf*EXP-1 [16]; *Pf*GAP45 [17], *Pf*GAP50 ([18]; kindly provided by Veronique Beiss, Fraunhofer IME Aachen, Germany), *Pf*AMA1 [19], alpha-tubulin (CST), and against the haemagglutinin (HA)-tag (Sigma-Aldrich). The generation of antisera against *Pf*WLP1 is described below.

### Parasite lines

For the study, either *P. falciparum* wild-type (WT) strain NF54 (ATCC) or the following *P. falciparum* knock-out parasites were used: *Pf*CCp1KO [13], *Pf*CCp4KO [12], *Pf*s230-delta1 and *Pf*s230-delta2 [20], *Pf*s48/45KO [21]. The cultures were cultivated with or without the selection drug pyrimethamine added to the culture medium (see below) in intervals of 3 weeks to remove potential revertants. The generation of the *Pf*WLP1-HA line is described below.

### Parasite culture

Asexual blood stages and gametocytes of *P. falciparum* WT strain NF54 or the above listed knock-out parasite lines were cultivated in vitro in human A^+^ erythrocytes as described [22]. The RPMI1640/HEPES medium (Gibco) was complemented with 10 % v/v heat-inactivated human serum, 50 µg/ml hypoxanthine (Sigma-Aldrich) and 10 µg/ml gentamicin (Gibco) and cultures were kept in an atmosphere of 5 % O_2_, 5 % CO_2_, and 90 % N_2_ at 37 °C. Human A^+^ erythrocyte sediment and serum were purchased from the Institute of Transfusion Medicine, University Hospital Aachen, Germany (PO No DKG-NT 9748). The erythrocyte and sera samples were pooled and the donors remained anonymous; the work on human blood was approved by the Ethics Commission of RWTH Aachen University. For cultivation of the knock-out parasite lines, pyrimethamine at a final concentration of 502 µM was added to the medium. To synchronize the asexual parasite blood stages, parasite cultures with 3–4 % ring stages were centrifuged, the pellet was resuspended in five times pellet’s volume of 5 % sorbitol (AppliChem)/ddH_2_O incubated for 10 min at room temperature (RT) [23]. The cells were washed once with RPMI medium to remove the sorbitol, diluted to 5 % v/v hematocrit with cell culture medium and further cultivated as described above. For enrichment of gametocytes, cultures were harvested and enriched by 80/65/50/35 % v/v Percoll gradients (GE Healthcare Life Sciences) as described [24] and parasites were collected at the 50/35 % v/v Percoll gradient interfaces. Gametogenesis was induced by incubating mature gametocyte cultures in 100 µM xanthurenic acid dissolved in 1 % v/v 0.5 M NH_4_OH/ddH_2_O for 15–30 min at RT.

### Diagnostic RT-PCR

To analyze the expression of the *pfwlp1* gene in asexual blood stages and gametocytes, total RNA was isolated from synchronized ring, trophozoite and schizont cultures, as well as enriched immature (stage II-IV), mature (stage V) and activated gametocytes (30 min post-activation) of WT strain NF54 using the Trizol reagent (Invitrogen) according to the manufacturer’s protocol. Following phenol/chloroform extraction and ethanol precipitation, RNA preparations were treated with RNase-free DNase I (Qiagen) to remove residual genomic DNA. RNA samples were analysed photometrically and showed A260/280 ratios higher than 2.1. The cDNA synthesis was carried out using the SuperscriptIII First-Strand Synthesis System (Invitrogen) with 2 µg of each RNA sample, following the manufacturer’s instructions. Transcript for *pfwlp1* (272 bp) was amplified in 25 cycles using *Pf*WLP1RT1 forward primer 5′-TGGGGGTTCCAAGAAGTA-3′ and *Pf*WLP1RT1 reverse primer 5′-CGCTTATGGCTATATCTTG-3′. To confirm purity of asexual blood stage and gametocyte samples, transcripts were amplified for *pfama1* (180 bp) using *Pf*AMA1RT1 forward primer 5′-GGATTATGGGTCGATGGA-3′ and *Pf*AMA1RT1 reverse primer 5′-GATCATACTAGCGTTCTT-3′ and for *pfccp2* (187 bp) using *Pf*CCp2RT1 forward primer 5′-TCGGATGGAGAATCCGTT-3′ and *Pf*CCp2RT1 reverse primer 5′-GTATCCCATGTCTTGTGA-3′. Amplification of *pfaldolase* (378 bp) using *Pf*AldolaseRT1 forward primer 5′-TAGATGGATTAGCAGAAAGATGC-3′ and *Pf*AldolaseRT1 reverse primer 5′-AGAAACCAACCATCTTGAGTAGTGG-3′ was used as loading control and to test for residual genomic DNA in the negative control without reverse transcriptase. PCR products were separated by 1.2 % w/v agarose gel electrophoresis.

### Recombinant protein expression

**Fig. 1:**
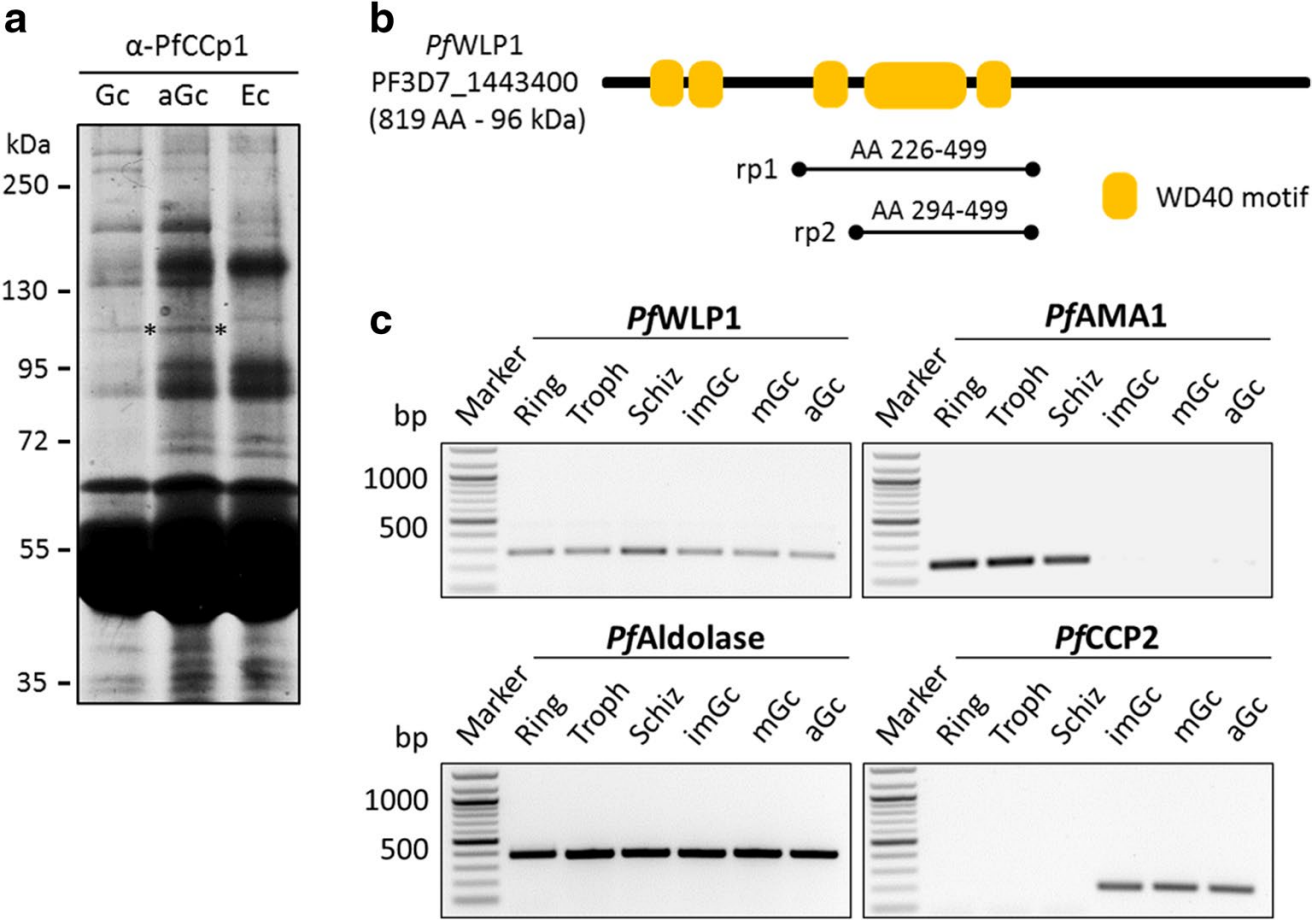
The WD40-repeat protein *Pf*WLP1 co-immunoprecipitates with *Pf*CCp1 and is transcribed in the *Plasmodium falciparum* blood stages. **a** Silver-stained SDS-PAGE of co-immunoprecipitated proteins from lysates of non-activated (Gc) and activated (aGc) gametocytes using anti-*Pf*CCp1 antisera as bait. Erythrocyte lysate (Ec) used as negative control. A protein running at ~100 kDa (*asterisks*) was identified by mass spectrometry as product of gene PF3D7_1443400, termed *Pf*WLP1. **b** Schematic of *Pf*WLP1. The five annotated WD40 motifs (*yellow boxes*) are represented. The underlined regions denote the recombinant proteins used for antisera generation. **c** Transcription of *pfwlp1* in the blood stages. Diagnostic RT-PCR was used to amplify *pfwlp1* transcript (272 bp) from ring stages (Ring), trophozoites (Troph), schizonts (Schiz), immature (imGc), mature (mGc) and activated gametocytes (aGC). Transcript analyses of *pfama1* (180 bp) and *pfccp2* (187 bp) were used to demonstrate purity of the asexual blood stage and gametocyte samples, respectively. Transcript analysis of *pfaldolase* (378 bp) was used for loading control. Data are representative of three independent experiments

Two recombinant proteins, *Pf*WLP1rp1 and *Pf*WLP1rp2, spanning AA226-499 and AA294-499, respectively (regions of recombinant proteins are indicated in Fig. 1b), were expressed as fusion proteins with a N-terminal glutathione-S-transferase (GST)-tag using the pGEX-4T-1 vector (Amersham Bioscience) for *Pf*WLP1rp1 or with a N-terminal maltose binding protein (MBP)-tag using the pIH902 vector [15] for *Pf*WLPrp2. Cloning was mediated by the addition of *Bam*HI/*Not*I (*Pf*WLP1rp1) and *Bam*HI/*Pst*I (*Pf*WLP1rp2) restriction sites to the ends of PCR-amplified gene fragments, using forward primer 5′-ATGGATCCATGATAGACCTAAATTATGTTAAATTG-3′ and reverse primer 5′- TAGCGGCCGCTTATCGTATTAGTGGTTTGTTTAAGCA-3′ for *Pf*WLP1rp1 and forward primer 5′- ATGGATCCAGTTCACGTTCAAATAAATCTGAT-3′ and reverse primer 5′-TACTGCAGTTATCGTATTAGTGGTTTGTTTAAGCA-3′ for *Pf*WLP1rp2 (restriction sites underlined). Recombinant proteins were expressed in BL21 (DE3) RIL cells according to the manufacturer’s protocol (Stratagene). Fusion proteins with respective sizes of ~58 kDa for *Pf*WLP1rp1 and ~65 kDa for *Pf*WLP1rp2 were purified via affinity chromatography from bacterial extracts using glutathione-sepharose (GE Healthcare) for *Pf*WLP1rp1 and amylose resin (New England Biolabs) for *Pf*WLP1rp2 according to the manufacturer’s protocols. The purity of the proteins was demonstrated by SDS-PAGE and Coomassie brilliant blue-staining (Thermo Fisher Scientific) according to the manufacturer’s protocol (Additional file 1).

### Generation of mouse antisera

Recombinant fusion proteins *Pf*WLP1rp1-GST and *Pf*WLP1rp2-MBP were purified by affinity chromatography as stated above. PBS buffer exchange was performed prior to immunization via filter centrifugation using Amicon Ultra 15 centrifugal filter units (Sigma Aldrich). Immune sera were generated by the immunization of 6 week-old female NMRI mice (Charles River Laboratories) with 100 µg recombinant protein emulsified in Freund’s incomplete adjuvant (Sigma-Aldrich) followed by a boost after 4 weeks. Mice were anesthetized 10 days after the boost by intraperitoneal injection of a mixture of ketamine and xylazine according to the manufacturer´s protocol (Sigma-Aldrich) followed by the collection of polyclonal immune sera via heart puncture. Immune sera of three mice immunized with the same antigen were pooled; sera of non-immunized mice (NMS) were collected for negative control in the experiments. The reactivity of the two antisera against the respective recombinant proteins was demonstrated via Western blotting as described below (Additional file 1). Experiments for the generation of antisera in mice were approved by the animal welfare committees of the government of Lower Franconia, Germany (ref. no. 55.2-2531.01-58/09), and of the District Council of Cologne, Germany (ref. no. 84-02.05.30.12.097 TVA).

### Reverse genetics plasmid construction

A reverse genetic construct aiming for gene disruption of the *pfwlp1* locus via single-crossover homologous recombination was generated using the vector pCAM-BSD [25–29]. A 404 bp fragment of the *pfwlp1* locus was amplified by PCR from *P. falciparum* WT strain NF54 genomic DNA with gene-specific forward primer 5′-ATGGATCCCCGATATAAATAACATAAGCTAC-3′ and reverse primer 5′-TAGCGGCCGCTTACATATTATCACTCTCTGAACAGTT-3′ introducing *Bam*HI/*Not*I restriction sites (underlined) in the PCR fragment, which included a stop codon. The PCR product was ligated to the *Bam*HI/*Not*I-cut pCAM-BSD vector. C′-terminal double HA-tagged *Pf*WLP1 was generated by inserting 417 bp of the homologous 3′-end of the coding gene sequence lacking a stop codon into the pCAM-BSD vector containing double HA-tag and 3′UTR from *P. berghei* DHFR-ts. The fragment of the 3′-end of the *pfwlp1* coding sequence was amplified by PCR from *P. falciparum* WT strain NF54 genomic DNA with gene-specific forward primer 5′-ATCTGCAGTATGTCAAATCATACTTTAACCAT-3′ and reverse primer 5′-TAGGATCCAAAAGCCACAAACGCCCA-3′ introducing *Pst*I/*Bam*HI restriction sites (underlined). The PCR product was ligated to the *Pst*I/*Bam*HI-cut pCAM-BSD HA tagged vector.

### Parasite transfection and genotype characterization

*Plasmodium falciparum* WT strain NF54 cultures with 4 % ring stages was electroporated with 60 µg of the respective plasmid DNA in transfection buffer as described [25–29]. Blastidicin (Invivogen) was added to a final concentration of 5.4 µM starting 4 h after transfection. Resistant parasites appeared three to 4 weeks after transfection. After 40–90 days of selection, the respective cultures were analysed for plasmid integration by diagnostic PCR. Genomic DNA of the transfected cultures was used as template in the diagnostic PCR and was isolated using the NucleoSpin Blood Kit (Macherey–Nagel) according to the manufacturer’s protocol. The following primers were used to investigate for vector integration and for the presence of episomal DNA: *Pf*WLP1-KO-5′ integration forward primer 5′-GGGTTCCAAGAAGTACATCAA-3′ (primer 1), *Pf*WLP1-KO-3′ integration reverse primer 5′-ACACCATCTCCTCCACCTGA-3′ (primer 2), *Pf*WLP1-HA-5′ integration forward primer 5′-AACAAATTAGAACCCACATGGTC-3′ (primer 1), *Pf*WLP1-HA-3′ integration reverse primer 5′-TTCCAGGAGGAACTCCAGTG-3′ (primer 2), pCAM-BSD forward primer 5′-TATTCCTAATCATGTAAATCTTAAA-3′ (primer 3) and pCAM-BSD reverse primer 5′-CAATTAACCCTCACTAAAG-3′ (primer 4). Regions of primers are indicated in Additional file 2A, C.

### Co-immunoprecipitation assay

Pellets of non-infected human erythrocytes or synchronized schizonts and enriched non-activated gametocytes of the *P. falciparum* WT strain NF54 or the *Pf*WLP1-HA line were resuspended in 0.5 % w/v saponin/0.5 % w/v NP40/PBS, homogenized and sonicated for 1 min as described [13]. Homogenate was pelleted at 16,000 g and the supernatant was pre-purified by incubation with 5 % v/v NMS followed by 20 µl protein G-beads (Santa Cruz Biotechnology) for 30 min each at 4 °C. After centrifugation at 3400*g*, the supernatant was incubated for 1 h at 4 °C with 5 % v/v mouse anti-*Pf*CCp1 antisera or rabbit antisera against the HA-tag, followed by incubation with 20 µl protein G-beads for 1 h or overnight. The beads were centrifuged, washed five times with PBS and mixed with an equal volume of SDS-loading buffer for SDS-PAGE. Precipitated proteins were analysed via Western blot as described below.

### Mass spectrometry

A co-immunoprecipitation assay on gametocyte lysate of the *P. falciparum* WT strain NF54 was conducted as described above using mouse antisera specific to *Pf*CCp1. The charge of anti-*Pf*CCp1 antisera used for co-immunoprecipitation was identical with the one used in a recently published co-immunoprecipitation study on the *Pf*CCp-based protein complexes of gametocytes [14]. Precipitated proteins were separated by SDS-PAGE followed by Silver staining using Pierce Silver Stain for Mass Spectrometry (Thermo Scientific) according to the manufacturer’s protocol. Lysate of non-infected erythrocytes was used as a negative control. Visible protein bands were cut out and an in-gel trypsin digest was performed prior to mass spectrometry analysis as described [30], followed by desalting and concentrating using ZipTips™ columns made from the reverse chromatography resins Poros and Oligo R3 (Applied Biosystems). Bound peptides were washed with 0.5 % w/v formic acid and eluted in 1 µl of 33 % v/v acetonitrile/0.1 % w/v trifluoroacetic acid solution saturated with α-cyano-4-hydroxycinnamic acid (Bruker Daltonics) onto a MALDI target plate and air dried before analysis in the Ultraflex-TOF TOF tandem mass spectrometer (Bruker Daltonics). Peptide mass fingerprint spectra were received in the reflectron positive mode with a pulsed extraction using ~100 laser shots. Spectra were obtained after an external calibration using reference peptides (Peptide mixture II, Bruker Daltonics). Monoisotopic masses were ascribed and processed using the software Biotools™ and FlexAnalysis™ (Bruker Daltonics) after internal calibration with trypsin autolysis peaks as internal standards (842.5100, 2211.1046 Da). Processed peptide mass fingerprints were submitted to the Mascot program [31] for searches against the non-redundant NCBI database with the following parameters: Taxonomy, *P.falciparum* and *Homo sapiens*; search all molecular masses and all isoelectric points; allow up to one missed proteolytic cleavage site and a peptide mass tolerance of 100 ppm. Cysteine carbamidomethylation was considered as a fixed modification and methionine oxidation as an optional modification in all the searches. Matches to human proteins were regarded as definite if the probability score was significant using the Mascot score with a p value <0.05.

### Western blot analysis

*Plasmodium falciparum* asexual blood stage parasites of WT strain NF54 or the *Pf*WLP1-HA line were harvested and treated with 0.15 % w/v saponin for erythrocyte lysis. Non-activated gametocytes of WT strain NF54 or of *Pf*WLP1-HA line were enriched by Percoll gradient purification as described above. Enriched activated gametocytes were collected at 30 min post-activation. The recombinant proteins *Pf*WLP1rp1-GST and *Pf*WLP1rp2-MBP were affinity-purified as described above. The cell pellets or recombinant proteins were resuspended in PBS and 2× SDS-PAGE loading buffer. Proteins were separated by SDS-PAGE and transferred to Hybond ECL nitrocellulose membrane (Amersham Biosciences) according to the manufacturer’s instructions. Non-specific binding was blocked by incubation of the membranes in Tris-buffered saline containing 5 % w/v skim milk and 1 % w/v bovine serum albumin fraction V, followed by immune recognition for 2 h at RT using mouse immune sera specific for *Pf*CCp1, *Pf*s230, *Pf*39, or rabbit antisera specific for the HA-tag or *Pf*AMA1. Afterwards membranes were washed, incubated for 1 h at RT with a goat anti-mouse or anti-rabbit alkaline phosphatase-conjugated secondary antibody (Sigma-Aldrich) and developed in a solution of nitroblue tetrazolium chloride (NBT) and 5-brom-4-chlor-3-indoxylphosphate (BCIP; Sigma-Aldrich). Scanned blots were processed using Adobe Photoshop CS software.

### Indirect immunofluorescence assay

**Fig. 2:**
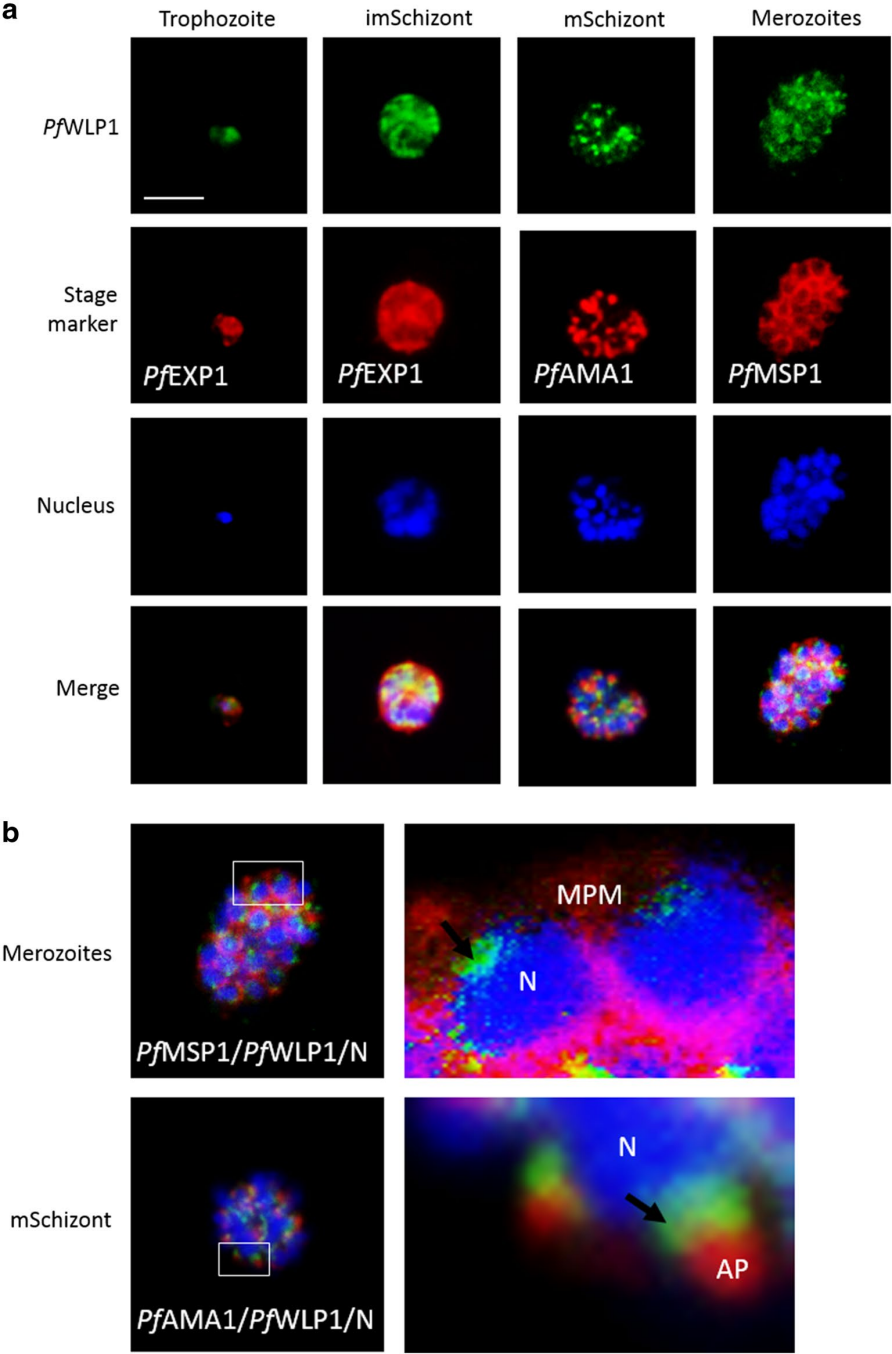
*Pf*WLP1 is expressed in the asexual blood stages of *Plasmodium falciparum.* **a** Expression of *Pf*WLP1 in the asexual blood stages. *Pf*WLP1 was immunolabelled with anti-*Pf*WLP1-rp2 antisera (*green*); the asexual blood stages were visualized with antisera against *Pf*EXP1, *Pf*AMA1 and *Pf*MSP1 (*red*). The parasite nuclei were highlighted by Hoechst nuclear stain (*blue*). ImSchizont, immature schizont; mSchizont, mature schizont. **b** Localization of *Pf*WLP1 underneath the apical pole of merozoites. Image enlargements (*right*) of mature schizonts (*left*), labelled with antibodies against *Pf*WLP1 (*green*) and *Pf*MSP1 or *Pf*AMA1 (*red*) and stained with Hoechst (*blue*). *AP* apical pole, *N* nucleus, *MPM* merozoite plasma membrane. *Bar* 5 µm. Data are representative of five independent experiments each

**Fig. 3:**
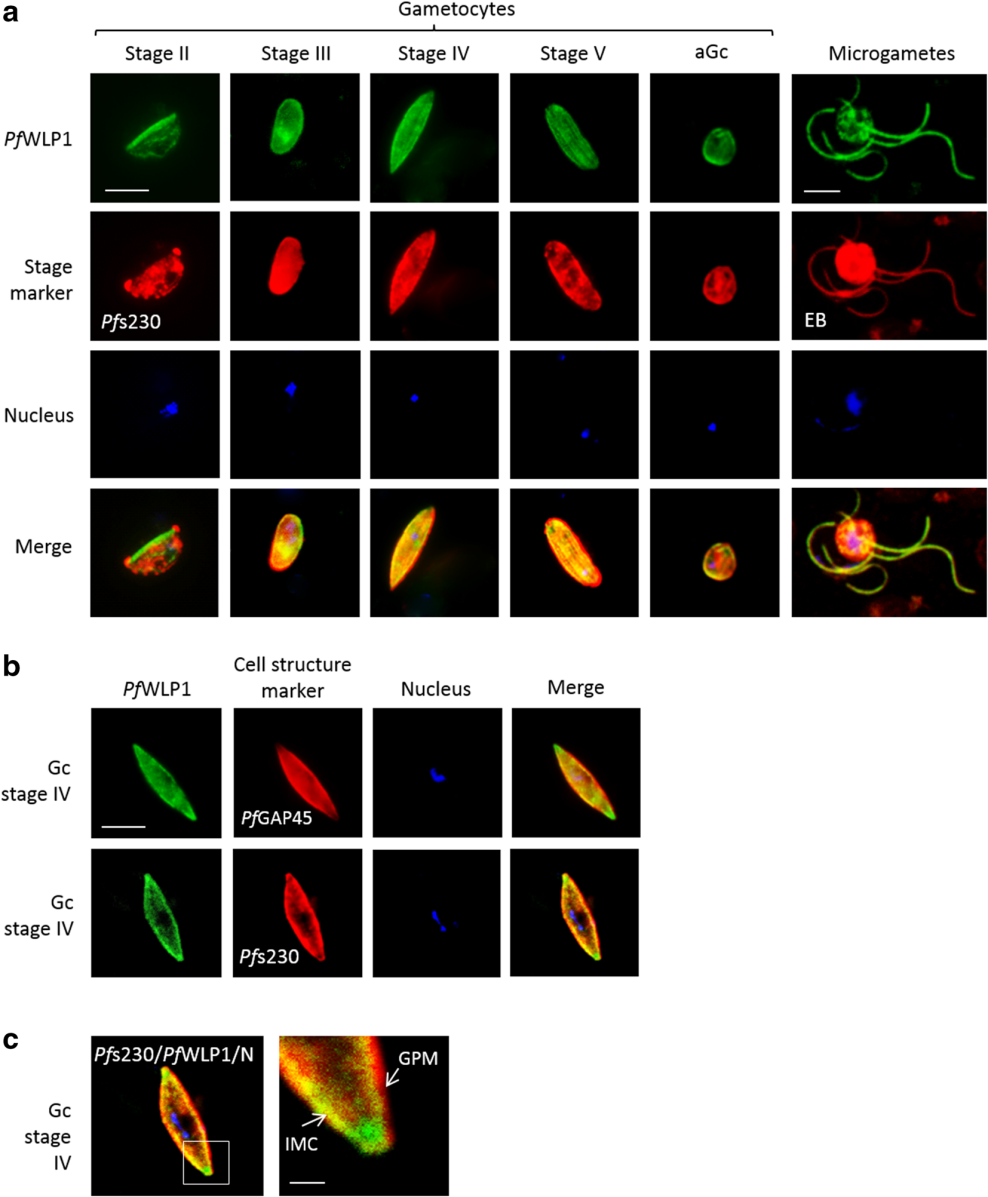
*Pf*WLP1 is expressed in the sexual stages of *Plasmodium falciparum.* **a** Expression of *Pf*WLP1 in non-activated and activated gametocytes. *Pf*WLP1 was immunolabelled with anti-*Pf*WLP1rp2 antisera (*green*); the sexual stages were visualized with anti-*Pf*s230 antisera or by Evans Blue (EB) counterstaining (*red*). The parasite nuclei were highlighted by Hoechst nuclear stain (in *blue*). **b** Co-localization of *Pf*WLP1 with distinct cell structures of gametocytes. *Pf*WLP1 was immunolabelled with anti-*Pf*WLP1rp2 antisera (*green*). The plasma membrane-associated adhesion protein *Pf*s230 and the inner membrane complex component *Pf*GAP45 were visualized using the respective rabbit antisera (*red*), nuclei were stained with Hoechst (*blue*). **c** Localization of *Pf*WLP1 underneath the gametocyte plasma membrane. Image enlargement (*right*) of a stage IV gametocyte, labelled with antibodies against *Pf*WLP1 (*green*) and *Pf*s230 (*red*), nuclei were stained with Hoechst (*blue*). *IMC* inner membrane complex, *GPM* gametocyte plasma membrane. *Bar* 5 µm (**a**, **b**), 1 μm (**c**). Data are representative of five independent experiments

**Fig. 4:**
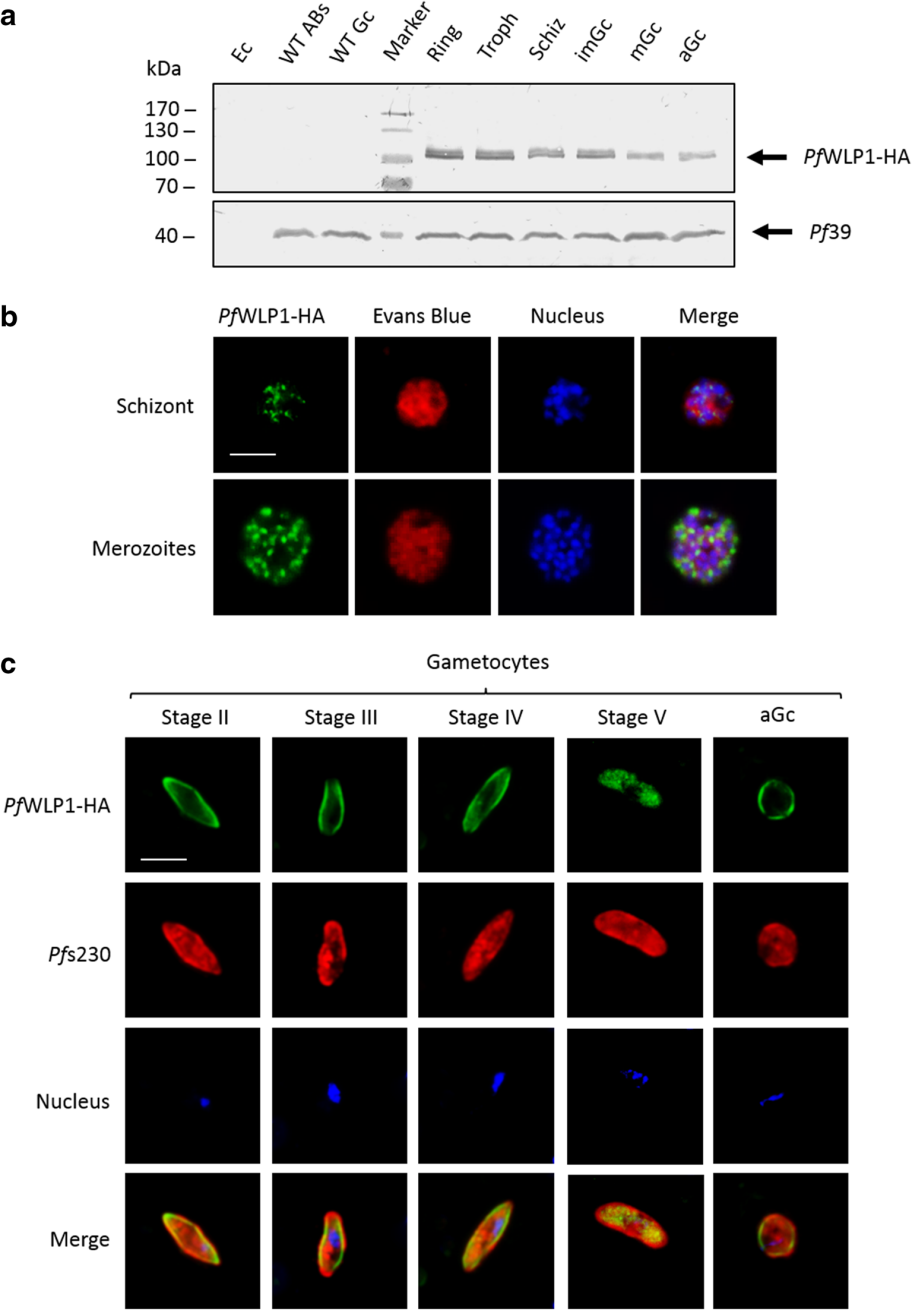
Expression of *Pf*WLP1-HA in asexual blood stages and gametocytes. **a** Detection of *Pf*WLP1-HA in parasite lysates. Western blot analysis of lysates from ring stages (Ring), trophozoites (Troph), schizonts (Schiz), immature gametocytes (imGc), mature gametocytes (mGc) and activated gametocytes (aGc) of line *Pf*WLP1-HA, using rabbit anti-HA antibody, detected *Pf*WLP1-HA migrating at the expected size of ~108 kDa. Lysates of WT asexual blood stages (WT ABs) and gametocytes (WT Gc) as well as of non-infected erythrocytes (Ec) was used as negative controls. Equal loading of lanes was confirmed by blotting with mouse antisera against *Pf*39 (~39 kDa). **b** *Pf*WLP1 expression in the *Pf*WLP1-HA asexual blood stages. *Pf*WLP1-HA was immunolabelled with anti-HA antisera (*green*); the asexual blood stages were visualized by Evans Blue counterstaining (*red*). The parasite nuclei were highlighted by Hoechst nuclear stain (*blue*). **c** *Pf*WLP1 expression in the *Pf*WLP1-HA non-activated and activated gametocytes. *Pf*WLP1-HA was immunolabelled with anti-HA antisera (*green*). The sexual stages were visualized with antisera against *Pf*s230 (*red*); nuclei were stained with Hoechst (*blue*). *Bar* 5 µm. Data are representative of two to five independent experiments

**Fig. 5:**
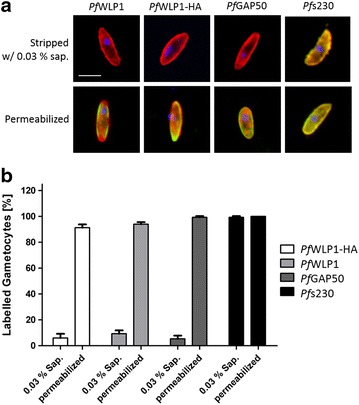
*Pf*WLP1 is intracellularly located in gametocytes. **a** Detection of plasma membrane-bound proteins. Gametocytes were either stripped from the enveloping erythrocyte and parasitophorous vacuole membranes by saponin-treatment prior to fixation or were membrane-permeabilized following fixation. The cells were immunolabelled with antibodies directed against *Pf*WLP1, *Pf*s230, *Pf*GAP50 or the HA-tag (*green*); the gametocytes were counterstained with Evans Blue (*red*). The parasite nuclei were highlighted by Hoechst nuclear stain (*blue*). *Bar* 5 µm. **b** Quantification of immunolabelling. A total number of 50 gametocytes were evaluated for immunolabelling as described in **a** in triplicate. Data are representative of three independent experiments

Parasite preparations for indirect immunofluorescence assays (IFAs) included mixed asexual blood stages and mixed gametocyte cultures as well as activated gametocyte-cultures at 15 min post-activation of WT strain NF54 or line *Pf*WLP-1-HA. Cell monolayers were air-dried on glass slides and subsequently fixed with 4 % w/v paraformaldehyde/PBS (pH 7.4) for 10 min at RT. For membrane permeabilization and blocking of non-specific binding sites, preparations were permeabilized with 0.1 % v/v Triton X-100/125 mM glycine (Carl Roth)/PBS at RT for 30 min, followed by blocking with 3 % w/v BSA/PBS for 1 h. To liberate live gametocytes from the enveloping RBC and parasitophorous vacuole membranes, gametocytes were treated with 0.05 % w/v saponin/medium for 3 min at 37 °C prior fixation, as described in Simon et al. [18]. In these cases, no further membrane permeabilization step was employed. Preparations were then incubated with polyclonal mouse antisera specific for *Pf*s230, *Pf*CCp1, *Pf*CCp4, and *Pf*WLP1 or with rabbit antisera directed against the HA-tag or *Pf*GAP50 at 37 °C for 2 h. NMS was used for negative control. Binding of primary antibody was detected by incubating the preparations with polyclonal Alexa Fluor 488-conjugated goat anti-mouse antibodies (Invitrogen Molecular Probes) at RT for 1 h. The different parasite stages or cell structures were either detected by double-labelling with stage-specific antibodies, i.e., polyclonal rabbit antisera directed against *Pf*MSP1, *Pf*AMA1, *Pf*EXP1, *Pf*s230, *Pf*GAP45, or alpha-tubulin, followed by incubation with polyclonal Alexa Fluor 594- or Alexa Fluor 633-conjugated goat anti-rabbit antibodies (Invitrogen Molecular Probes), or the cells were counterstained with 0.05 % w/v Evans Blue (Sigma-Aldrich)/PBS for 1 min at RT. Antisera dilutions of 1:50 to 1:1000 were used. The parasite nuclei were highlighted by incubating the specimens with Hoechst nuclear stain 33342 (Invitrogen Molecular Probes) for 1 min at RT. Labelled specimens were examined by confocal laser scanning microscopy using a Zeiss LSM 510 microscope (Additional file 3) or a Leica TCS SP5 DM6000 CFS microscope, equipped with a 20× 1.0 NA water immersion objective (Additional file 4B). Dyes were excited using the 488 nm line of an argon laser and a 633 nm HeNe laser. Otherwise the labelled specimens were investigated using an Olympus BX41 fluorescence microscope in combination with a ProgRes Speed XT5 camera (Fig. 2a, b), or using a Leica DM5500 B fluorescence microscope in combination with a Leica DFC365 FX camera (Figs. 2a–c, 3a–c, 4b, c, 5a; Additional files 5, 6, 7A). Digital images were processed using Adobe Photoshop CS software. *Pf*WLP1 expression was quantified by determining the percentage of immune-labelled gametocytes (n = 100) in three individual experiments. Labelling of non-permeabilized and permeabilized gametocytes (n = 50) was determined in triplicate.

## Results

### A WD40-repeat protein interacts with the *Pf*CCp-based protein complex of gametocytes

Previous work demonstrated that the six secreted *Pf*CCp proteins assemble to multimeric protein complexes that locate in the parasitophorous vacuole associated to the plasma membrane of gametocytes. The *Pf*CCp-based protein complex is bound to the secreted large 6-cys protein *Pf*s230, which itself is linked to the plasma membrane via an interaction with the GPI-anchored *Pf*s48/45 [11–14, 32, 33]. It was the initial aim of this study to identify potential additional proteins associated with the *Pf*CCp-based protein complex. For this purpose co-immunoprecipitation assays were performed using anti-*Pf*CCp1 antisera as bait. Proteins precipitated from lysates of non-activated and activated gametocytes were separated by SDS-PAGE followed by silver staining; precipitates from lysates of non-infected erythrocytes were used as a negative control. By silver staining a protein running at a molecular weight of ~100 kDa was identified in the lysates of non-activated and activated gametocytes, which was absent in the erythrocyte lysate (Fig. 1a). The protein bands were excised from the gel and subjected to mass spectrometry. Peptides were assigned to a yet unknown protein of 96 kDa encoded by gene PF3D7_1443400 [34, 35]. Annotations indicated the presence of five WD40 motifs (Fig. 1b); thus the protein was termed *P. falciparum* WD40-repeat protein-like protein, *Pf*WLP1.

Computational homology searches revealed that homologues of *Pf*WLP1 are encoded in the genomes of the rodent malaria parasites *Plasmodium berghei*, *Plasmodium chabaudi* and *Plasmodium yoelii*, in the ape-specific species *Plasmodium cynomolgi* and *Plasmodium reichenowi* and in the human malaria parasites *Plasmodium knowlesi* and *Plasmodium vivax* (Additional file 2). Contrary to all other *pwlp1* genes, *pvwlp1* of *P. vivax* encodes for six WD40 motifs.

To confirm expression of *pfwlp1* in gametocytes, diagnostic RT-PCR was conducted, using cDNA obtained from enriched immature (stage II–IV) and mature (stage V) gametocytes and from gametocytes at 30 min post-activation, further cDNA samples from purified ring stages, trophozoites and schizonts were tested for *pfwlp1* expression. Diagnostic RT-PCR demonstrated transcript abundance for *pfwlp1* in all of the asexual blood and gametocyte stages, which increased in schizonts (Fig. 1c). Quality of the cDNA samples was verified by monitoring transcripts of stage-specific marker proteins, i.e., *pfama1* for the asexual blood stages and *pfccp2* for gametocytes. Amplification of transcript encoding for the housekeeping protein *pfaldolase* was used as an equal-loading control (Fig. 1c). No PCR products were amplified from mock-treated RNA samples lacking reverse transcriptase, indicating that genomic DNA was absent (Additional file 3A).

### *Pf*WLP1 accumulates underneath the micronemes of merozoites and the gametocyte plasma membrane

To investigate *Pf*WLP1 protein expression in the asexual blood and gametocyte stages, mouse antisera against two bacterially expressed peptides of *Pf*WLP1, i.e., *Pf*WLP1rp1 and *Pf*WLP1rp2 (see Fig. 1b) were generated. The *Pf*WLP1 expression pattern was determined via IFA, using anti-*Pf*WLP1rp2 antisera. In trophozoites, highlighted by MSP1-labelling, a minor expression of *Pf*WLP1 was observed, while the *Pf*WLP1 signal increased during schizont maturation (Fig. 2a). In mature schizonts *Pf*WLP1 accumulated underneath the apical pole of the forming merozoites, highlighted by labelling of the microneme transmembrane protein *Pf*AMA1.

The localization of *Pf*WLP1 in the maturing schizonts was investigated in more detail. Image enlargements of co-labelling experiments using anti-*Pf*MSP1 antisera to highlight the parasite plasma membrane depict *Pf*WLP1 in the perinuclear region (Fig. 2b); co-labelling experiments using anti-*Pf*AMA1 antisera demonstrated *Pf*WLP1 accumulation between apical pole and nucleus. Laser scanning confocal microscopy was used to follow the redistribution of *Pf*WLP1 during schizont maturation and revealed that *Pf*WLP1 is originally found underneath the *Pf*MSP1-positive plasma membrane of the immature schizont, from where it redistributes to focal spots close to the apical pole, once the merozoites form (Additional file 3B).

*Pf*WLP1 is further abundantly expressed in the developing gametocytes, highlighted by *Pf*s230-labelling; here *Pf*WLP1 labelling was found to be both intracellular and plasma membrane-associated (Fig. 3a). A total of 94 ± 3 % of *Pf*s230-labelled gametocytes were also positive for *Pf*WLP1 (n = 100, three individual experiments), indicating that *Pf*WLP1 is expressed by gametocytes of both genders. In mature gametocytes, the peripheral labelling often disappeared and the presence of *Pf*WLP1 was then restricted to the cytosol. *Pf*WLP1 expression was further detected in macro- and microgametes (Fig. 3a). When NMS was used in the IFAs, no labelling was detected in the asexual blood stages or gametocytes (Additional file 4A). When anti-*Pf*WLP1rp1 antisera were used in the IFAs, a similar labelling pattern was observed in schizonts and gametocytes (Additional file 4B).

Because in developing gametocytes the majority of *Pf*WLP1 is located close to the plasma membrane, co-localization experiments were performed, using antisera against proteins located in close proximity to the plasma membrane, i.e., the peripheral protein *Pf*s230, the inner membrane complex protein *Pf*GAP45 and the cytoskeletal protein alpha-tubulin. The IFAs demonstrated that *Pf*WLP1 is localized in close proximity to the inner membrane complex and image enlargement revealed that *Pf*WLP1 is located underneath the *Pf*s230-positive plasma membrane (Fig. 3b, c). Further, *Pf*WLP1 co-localized with alpha-tubulin in the maturing schizonts and gametocytes (Additional file 5). Noteworthy, in the mature stage V gametocytes, when the tubulin network underneath the plasma membrane disassembles ([36], reviewed in [37]), the rim-associated *Pf*WLP1 labelling also disappeared. Because *Pf*WLP1 is also expressed in lines *Pf*CCp1KO, *Pf*CCp4KO, *Pf*s230-delta1, *Pf*s230-delta2, and *Pf*s48/45KO (Additional file 6), the expression of *Pf*WLP1 is not dependent on the presence of any of the components of the *Pf*CCp-based multimeric protein complex.

### *Pf*WLP1 is crucial for erythrocytic schizogony

For functional studies the *pfwlp1* gene locus was targeted for disruption via single cross-over homologous recombination, using the pCAM-BSD vector [25–29]. The *pfwlp1*-*KO* vector contained an insert encoding the N-terminal portion of the protein, as well as a cassette conferring resistance to blasticidin. Integration into the respective *pfwlp1* gene would result in a disrupted (pseudo-diploid) locus (Additional file 7A).

The pCAM-BSD-based *pfwlp1*-KO vector was electroporated into ring-stage parasites and populations of blasticidin-resistant parasites were obtained. However, these parasites contained only non-integrated episomes and no integration of the respective *pfwlp1*-*KO* vector was detected by diagnostic PCR (Additional file 7B) even after prolonged culturing (24 weeks).

To verify that the *pfwlp1* locus was accessible for recombination, a pCAM-BSD-based vector that contained an insert encoding the 3′-end of *Pf*WLP1 fused to the sequence encoding a double HA-tag, followed by the 3′-untranslated region from the *P. bergheidhfr*-*ts* gene, was generated (Additional file 7C). Integration of the vector would result in a complete *pfwlp1* gene followed by a 3′-located HA-sequence. This recombination is predicted not to cause loss of function of the gene product, but to generate a functional protein.

The *pfwlp1*-*HA* vector was electroporated into ring-stage cultures and treated with blasticidin as described above. DNA was isolated from blasticidin-resistant HA-tag vector populations ~6–8 weeks following electroporation and PCR revealed that integration of the vector had occurred. Following clonal dilution, the *pfwlp1*-*HA* clone 1A7 was isolated (Additional file 7D).

Expression of HA-tagged *Pf*WLP1 was confirmed via Western blotting, using anti-HA antibodies. In the *Pf*WLP1-HA line, HA-tagged *Pf*WLP1 was observed in lysates of enriched ring stages, trophozoites and schizonts, as well as in cultures of immature and mature gametocytes and in gametocytes at 30 min post-activation. In all lysates, *Pf*WLP1-HA was running at a predicted molecular weight of ~108 kDa (*Pf*WLP1 ~96 kDa; HA-tag ~12 kDa) (Fig. 4a). No protein bands were detected when lysates of non-infected erythrocytes or lysates of WT asexual blood stages and gametocytes were immunoblotted with the anti-HA antibody. Immunolabelling with anti-*Pf*39 was used as a loading control (Fig. 4a).

IFAs confirmed the expression of *Pf*WLP1-HA in schizonts and merozoites of the *Pf*WLP1-HA line, and the protein initially localized underneath the plasma membrane and then accumulated underneath the apical poles of the forming merozoites (Fig. 4b). Further, *Pf*WLP1-HA was found in the developing gametocytes of the *Pf*WLP1-HA line (Fig. 4c). Consistent with the *Pf*WLP1 location in WT parasites, the HA-tagged protein was present both in the cytoplasm and associated to the gametocyte plasma membrane. In mature gametocytes, the peripheral labelling disappeared. *Pf*WLP1-HA was further observed associated to the surface of the emerging gametes (Fig. 4c). IFAs further confirmed a co-labelling, when antibodies directed against *Pf*WLP1 (*Pf*WLP1rp2) and the HA-tag were used (Additional file 8A). Also, *Pf*WLP1-HA was detectable, when the protein was immunoprecipitated from a lysate of the *Pf*WLP1-HA line, using anti-*Pf*WLP1rp2 antisera, and when the precipitate was subsequently immunoblotted with anti-HA antibody (Additional file 8B).

To confirm that *Pf*WLP1 is an intracellular protein that localizes underneath the gametocyte plasma membrane, live gametocytes were collected and liberated from the enveloping RBC and parasitophorous vacuole membranes via mild saponin-treatment as previously described [18]. The gametocytes were subsequently fixed with paraformaldehyde, one part of the fixed cells was then membrane-permeabilized, while the other portion was not membrane-permeabilized. Subsequent IFAs showed a labelling for the peripheral plasma membrane-bound protein *Pf*s230 in non-permeabilized gametocytes, but neither a labelling for the inner membrane complex protein *Pf*GAP50 or for *Pf*WLP1 was observed these cells, indicating that *Pf*WLP1, like *Pf*GAP50, is located intracellularly and thus not accessible for the antibodies in the IFAs. Also no labelling was detected for the HA-tag, when saponin-treated non-membrane permeabilized gametocytes of the *Pf*WLP1-HA line were used (Fig. 5a). Furthermore, quantification of non-permeabilized gametocytes showed that only minor proportions are labelled for the intracellular protein *Pf*GAP50 (5.3 ± 2.4 %) or for *Pf*WLP1 (9.3 ± 2.5 %) and *Pf*WLP1-HA (6 ± 3.2 %). In contrast 99.3 ± 0.94 % of non-permeabilized gametocytes showed labelling for the membrane-bound protein *Pf*s230 (n = 50; in triplicate) (Fig. 5b). When the membranes were permeabilized and thus were accessible for the antibodies, all of the gametocytes labelled for the respective proteins (Fig. 5a, b).

### *Pf*WLP1 associates with components of adhesion protein complexes

**Fig. 6:**
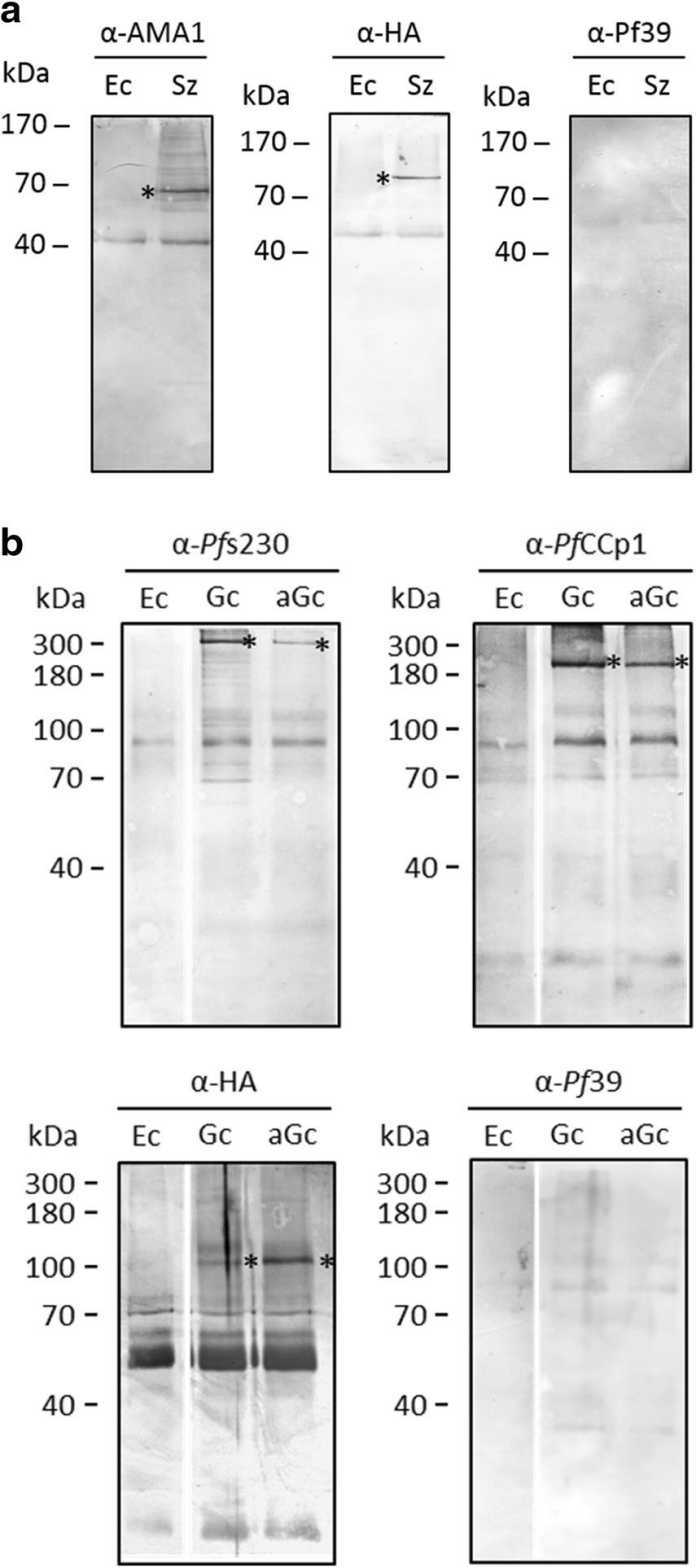
*Pf*WLP1 associates with cell adhesion proteins of merozoites and gametocytes. **a** Co-immunoprecipitation of *Pf*WLP1 with *Pf*AMA1. Lysates of enriched schizonts of line *Pf*WLP1-HA were immunoprecipitated with anti-HA antisera, followed by Western blot analysis using antisera directed against *Pf*AMA1 (~72 kDa). **b** Co-immunoprecipitation of *Pf*WLP1 with *Pf*s230 and *Pf*CCP1. Lysates of non-activated and activated gametocytes of line *Pf*WLP1-HA were immunoprecipitated with anti-HA antisera, followed by Western blot analysis using antisera directed against *Pf*s230 or *Pf*CCp1 (360 and 185 kDa, respectively). Immunoblotting with anti-HA antisera confirmed the presence of *Pf*WLP1-HA (~108 kDa) in all precipitates. A smeared protein band migrating at ~55 kDa resembled the heavy chain of the rabbit antibody. Erythrocyte lysate (Ec) and immunoblotting with mouse antisera directed against *Pf*39 (39 kDa) were used as a negative controls. *Asterisks* indicate the precipitated proteins. Data are representative of two to three independent experiments

In a final set of experiments the *Pf*WLP1-HA-expressing line was used to investigate, if *Pf*WLP1 is part of larger adhesion protein complexes of merozoites and gametocytes. Firstly, *Pf*WLP1-HA was immunoprecipitated from schizont-enriched lysate, using anti-HA antibody. The precipitate was subjected to SDS-PAGE followed by Western blotting. Immunoblotting detected *Pf*AMA1 (~72 kDa) in the precipitate, when the respective antibody was used (Fig. 6a). Subsequently, lysates of enriched gametocytes and of gametocytes at 30 min post-activation were used in the assays. By means of immunoblotting, *Pf*s230 and *Pf*CCp1 (~363 and 185 kDa, respectively) were detected in the precipitates (Fig. 6b). Immunoblotting with anti-HA antibody verified that the HA-tagged *Pf*WLP1 was present in the precipitates, which was running with a molecular weight of ~108 kDa. Immunoblotting with anti-*Pf*39 antibody, which was used as a negative control in both experiments, did not detect any protein bands (Fig. 6a, b). Neither were protein bands specific for *Pf*AMA1, *Pf*s230, *Pf*CCp1 or *Pf*WLP1-HA detected in lysates of non-infected erythrocytes (Fig. 6a, b). The co-immunoprecipitation data indicate that *Pf*WLP1 associates with components of adhesion protein complexes of merozoites and gametocytes.

## Discussion

WD40-repeat proteins belong to one of the most abundant protein classes in eukaryotic organisms, involved in a variety of cellular processes, such as signal transduction, cytoskeleton assembly, or cell cycle control. They all share a common function by acting as a scaffold for protein–protein interactions, thereby mediating the formation of protein complexes and in consequence coordinating downstream events ([38, 39], reviewed in [40, 41]).

The WD40 repeats are short repeating ~40–66 amino acid motifs, terminated by an eponymous tryptophan-aspartate (WD) dipeptide [38, 39]. Usually seven or multiple of seven WD40-repeats compose a WD40 domain, which is folded into a seven-bladed ß-propeller with a funnel-like shape, serving with the circumference as three main interaction sites. WD40 domains can act as a large interaction platform in cellular interaction networks; WD40-repeat proteins can thus be involved in various multi-protein complexes with different functions. Noteworthy, each WD40-repeat protein can possess multiple functions depending on its direct and indirect interactions partners ([42, 43]; reviewed in [40]).

A recent in silico analysis of the *P. falciparum* genome revealed 80 putative WD40-repeat proteins; these included 15 proteins specific to the genus *Plasmodium* [44]. In the same study, protein–protein interaction network analyses indicated more than 1900 potential interactions, and only nine of the plasmodial WD40-repeat proteins appear to be stage-specific.

The majority of plasmodial WD40-repeat proteins are, to date, of unknown functions and only two of them, *Pf*Sec13 and *Pf*RACK, have hitherto been studied in detail. In humans, Sec13 is known to be involved in the biogenesis of COPII-coated vesicles at the endoplasmic reticulum, whereas it also is an essential component in the nuclear pore complex [45]. *Pf*Sec13 has been described as an unusual component of the parasite nuclear pore complex involved in several putative cellular processes, such as chromatin regulation or vesicle biogenesis [46]. Another WD40-repeat protein characterized in *Plasmodium* is the receptor for activated C kinases (RACK) [47]. In humans, RACK proteins act as a scaffold for activated protein kinase C and other proteins, thereby stabilizing the active enzymes and increasing the substrate phosphorylation, and are further involved in integrin-dependent cell migration [48, 49]. In *P.falciparum*, *Pf*RACK is expressed during the erythrocytic replication cycle, where it is exported into the erythrocyte cytosol and able to inhibit mammalian Ca^2+^ signals thereby utilizing the host cell signalling machinery [47, 50].

The here-described *Pf*WLP1 is expressed in the maturing schizonts, where it is initially found underneath the plasma membrane and then relocalizes underneath the micronemes, once the merozoites have formed. *Pf*WLP1 is further expressed in the developing gametocytes, where it accumulates at the sub-pellicular region. Some of the complex partners of *Pf*WLP1 were identified, which in merozoites includes the micronemal transmembrane protein *Pf*AMA1, while in gametocytes *Pf*WLP1 associates with *Pf*s230 and *Pf*CCp1, two components of the *Pf*CCp-based protein complex. Further, *Pf*WLP1, which lacks any signal peptide, is an intracellular protein. For *Pf*AMA1, a direct interaction between *Pf*WLP1 and the C-terminal intracellular part of the transmembrane protein might be possible. The interaction between *Pf*WLP1 and peripheral proteins *Pf*s230 and *Pf*CCp1, though, has to be indirect and would have to be mediated by yet unknown membrane-spanning proteins. In view of the data that *Pf*WLP1 is found in close proximity with sub-pellicular proteins like *Pf*GAP45, *Pf*GAP50 or tubulin, larger protein complexes containing *Pf*WLP1 might connect adhesion proteins like *Pf*AMA1 or the *Pf*CCp proteins with structural elements of the parasite, e.g. the inner membrane complex or the cytoskeleton. Molecular connections between plasma membrane-associated adhesion proteins and structural elements are already known for the actin-myosin motor complex of the parasite invasive stages, where aldolase connects distinct transmembrane proteins of the TRAP family with the actin-myosin motor, while *Pf*GAP45/*Pf*GAP50 connect the actin-myosin motor with the outer membrane of the inner membrane complex (reviewed in [7]).

## Conclusion

This is the first report on a plasmodial WD40-repeat protein associating with cell adhesion protein complexes of the *P. falciparum* blood stages. The here presented data lead to the hypothesis that *Pf*WLP1 is involved in the stability or anchoring of membrane-linked adhesion protein complexes in schizonts and gametocytes, which, via larger protein complexes, might be connected to sub-pellicular or cytoskeletal elements of the parasite. Further analysis of the function of *Pf*WLP1 might reveal the detailed mode of action and deepen the knowledge on the role of WD40-repeat proteins in malaria parasites.

## Additional files

10.1186/s12936-015-0967-x Purity of the recombinant *Pf*WLP1 proteins and reactivity of the respective antisera.
10.1186/s12936-015-0967-x Homology analyses of *P*WLP1 proteins.
10.1186/s12936-015-0967-x RT-PCR genomic DNA control and *Pf*WLP1 relocalization in maturing schizonts.
10.1186/s12936-015-0967-x IFA negative and *Pf*WLP1rp1 antibody controls.
10.1186/s12936-015-0967-x Co-labelling of *Pf*WLP1 with alpha-tubulin.
10.1186/s12936-015-0967-x Expression of *Pf*WLP1 in WT and knock-out gametocytes.
10.1186/s12936-015-0967-x Strategy for *pfwlp1* gene locus disruption and generation of a HA-tagged *Pf*WLP1 parasite line.
10.1186/s12936-015-0967-x Co-detection of *Pf*WLP1-HA using anti-HA and anti-*Pf*WLP1 antibodies.

## References

[1] Baum J, Richard D, Healer J, Rug M, Krnajski Z, Gilberger T-W (2006). A conserved molecular motor drives cell invasion and gliding motility across malaria life cycle stages and other apicomplexan parasites. J Biol Chem.

[2] Kadekoppala M, Holder A (2010). Merozoite surface proteins of the malaria parasite: the MSP1 complex and the MSP7 family. Int J Parasitol.

[3] Cowman AF, Berry D, Baum J (2012). The cellular and molecular basis for malaria parasite invasion of the human red blood cell. J Cell Biol.

[4] Lin CS, Uboldi AD, Marapana D, Czabotar PE, Epp C, Bujard H (2014). The merozoite surface protein 1 complex Is a platform for binding to human Erythrocytes by *Plasmodium falciparum*. J Biol Chem.

[5] Alexander DL, Arastu-Kapur S, Dubremetz J-F, Boothroyd JC (2006). *Plasmodium falciparum* AMA1 binds a rhoptry neck protein homologous to TgRON4, a component of the moving junction in Toxoplasma gondii. Eukaryot Cell.

[6] Lamarque M, Besteiro S, Papoin J, Roques M, Vulliez-Le Normand B, Morlon-Guyot J (2011). The RON2-AMA1 interaction is a critical step in moving junction-dependent invasion by apicomplexan parasites. PLoS Pathog.

[7] Boucher LE, Bosch J (2015). The apicomplexan glideosome and adhesins—Structures and function. J Struct Biol.

[8] Kuehn A, Simon N, Pradel G (2010). Family members stick together: multi-protein complexes of malaria parasites. Med Microbiol Immunol.

[9] Pradel G (2007). Proteins of the malaria parasite sexual stages: expression, function and potential for transmission blocking strategies. Parasitology.

[10] Pradel G, Hayton K, Aravind L, Iyer LM, Abrahamsen MS, Bonawitz A (2004). A multidomain adhesion protein family expressed in *Plasmodium falciparum* is essential for transmission to the mosquito. J Exp Med.

[11] Pradel G, Wagner C, Mejia C, Templeton TJ (2006). *Plasmodium falciparum*: co-dependent expression and co-localization of the PfCCp multi-adhesion domain proteins. Exp Parasitol.

[12] Scholz SM, Simon N, Lavazec C, Dude M-A, Templeton TJ, Pradel G (2008). PfCCp proteins of *Plasmodium falciparum*: gametocyte-specific expression and role in complement-mediated inhibition of exflagellation. Int J Parasitol.

[13] Simon N, Scholz SM, Moreira CK, Templeton TJ, Kuehn A, Dude M-A (2009). Sexual stage adhesion proteins form multi-protein complexes in the malaria parasite *Plasmodium falciparum*. J Biol Chem.

[14] Simon N, Kuehn A, Williamson KC, Pradel G (2015). Adhesion protein complexes of malaria gametocytes assemble following parasite transmission to the mosquito. Parasitol Int.

[15] Williamson KC, Keister DB, Muratova O, Kaslow DC (1995). Recombinant Pfs230, a *Plasmodium falciparum* gametocyte protein, induces antisera that reduce the infectivity of *Plasmodium falciparum* to mosquitoes. Mol Biochem Parasitol.

[16] Günther K, Tümmler M, Arnold HH, Ridley R, Goman M, Scaife JG (1991). An exported protein of *Plasmodium falciparum* is synthesized as an integral membrane protein. Mol Biochem Parasitol.

[17] Jones ML, Cottingham C, Rayner JC (2009). Effects of calcium signaling on *Plasmodium falciparum* erythrocyte invasion and post-translational modification of gliding-associated protein 45 (PfGAP45). Mol Biochem Parasitol.

[18] Simon N, Lasonder E, Scheuermayer M, Kuehn A, Tews S, Fischer R (2013). Malaria parasites co-opt human factor H to prevent complement-mediated lysis in the mosquito midgut. Cell Host Microbe.

[19] Boes A, Spiegel H, Edgue G, Kapelski S, Scheuermayer M, Fendel R (2015). Detailed functional characterization of glycosylated and nonglycosylated variants of malaria vaccine candidate PfAMA1 produced in Nicotiana benthamiana and analysis of growth inhibitory responses in rabbits. Plant Biotechnol J.

[20] Eksi S, Stump A, Fanning SL, Shenouda MI, Fujioka H, Williamson KC (2002). Targeting and sequestration of truncated Pfs230 in an intraerythrocytic compartment during *Plasmodium falciparum* gametocytogenesis. Mol Microbiol.

[21] Van Dijk MR, Janse CJ, Thompson J, Waters AP, Braks JA, Dodemont HJ (2001). A central role for P48/45 in malaria parasite male gamete fertility. Cell.

[22] Ifediba T, Vanderberg J (1981). Complete in vitro maturation of *Plasmodium falciparum* gametocytes. Nature.

[23] Lambros C, Vanderberg JP (1979). Synchronization of *Plasmodium falciparum* erythrocytic stages in culture. J Parasitol.

[24] Kariuki MM, Kiaira JK, Mulaa FK, Mwangi JK, Wasunna MK, Martin SK (1998). *Plasmodium falciparum*: purification of the various gametocyte developmental stages from in vitro-cultivated parasites. Am J Trop Med Hyg.

[25] Sidhu ABS, Valderramos SG, Fidock DA (2005). pfmdr1 mutations contribute to quinine resistance and enhance mefloquine and artemisinin sensitivity in *Plasmodium falciparum*. Mol Microbiol.

[26] Dorin-Semblat D, Quashie N, Halbert J, Sicard A, Doerig C, Peat E (2007). Functional characterization of both MAP kinases of the human malaria parasite *Plasmodium falciparum* by reverse genetics. Mol Microbiol.

[27] Agarwal S, Kern S, Halbert J, Przyborski JM, Baumeister S, Dandekar T (2011). Two nucleus-localized CDK-like kinases with crucial roles for malaria parasite erythrocytic replication are involved in phosphorylation of splicing factor. J Cell Biochem.

[28] Solyakov L, Halbert J, Alam MM, Semblat J-P, Dorin-Semblat D (2011). Global kinomic and phospho-proteomic analyses of the human malaria parasite *Plasmodium falciparum*. Nat Commun.

[29] Kern S, Agarwal S, Huber K, Gehring AP, Strödke B, Wirth CC (2014). Inhibition of the SR protein-phosphorylating CLK kinases of *Plasmodium falciparum* impairs blood stage replication and malaria transmission. PLoS One.

[30] Hellman U, Wernstedt C, Góñez J, Heldin CH (1995). Improvement of an “In-Gel” digestion procedure for the micropreparation of internal protein fragments for amino acid sequencing. Anal Biochem.

[31] Mascot database (http://www.matrixscience.com).

[32] Kumar N (1987). Target antigens of malaria transmission blocking immunity exist as a stable membrane bound complex. Parasite Immunol.

[33] Kumar N, Wizel B, Nirbhay K, Wizel B (1992). Further characterization of interactions between gamete surface antigens of *Plasmodium falciparum*. Mol Biochem Parasitol.

[34] PlasmoDB Plasmodium Genomics Resource (http://www.PlasmoDB.org).

[35] Aurrecoechea C, Brestelli J, Brunk BP, Dommer J, Fischer S, Gajria B (2009). PlasmoDB: a functional genomic database for malaria parasites. Nucleic Acids Res.

[36] Dearnley MK, Yeoman JA, Hanssen E, Kenny S, Turnbull L, Whitchurch CB (2012). Origin, composition, organization and function of the inner membrane complex of *Plasmodium falciparum* gametocytes. J Cell Sci.

[37] Dixon MWA, Dearnley MK, Hanssen E, Gilberger T, Tilley L (2012). Shape-shifting gametocytes: how and why does *P. falciparum* go banana-shaped?. Trends Parasitol.

[38] Van der Voorn L, Ploegh HL (1992). The WD-40 repeat. FEBS Lett.

[39] Neer E, Schmidt C, Nambudripad R, Smith T (1994). The ancient regulatory-protein family of WD-repeat proteins. Nature.

[40] Stirnimann CU, Petsalaki E, Russell RB, Müller CW (2010). WD40 proteins propel cellular networks. Trends Biochem Sci.

[41] Xu C, Min J (2011). Structure and function of WD40 domain proteins. Protein Cell.

[42] Collins SR, Kemmeren P, Zhao X-C, Greenblatt JF, Spencer F, Holstege FCP (2007). Toward a comprehensive atlas of the physical interactome of *Saccharomyces cerevisiae*. Mol Cell Proteomics.

[43] Yu H, Braun P, Yildirim MA, Lemmens I, Venkatesan K, Sahalie J (2008). High-quality binary protein interaction map of the yeast interactome network. Science.

[44] Chahar P, Kaushik M, Gill SS, Gakhar SK, Gopalan N, Datt M (2015). Genome-wide collation of the *Plasmodium falciparum* WDR protein superfamily reveals malarial parasite-specific features. PLoS One.

[45] Enninga J, Levay A, Fontoura BMA (2003). Sec13 shuttles between the nucleus and the cytoplasm and stably interacts with Nup96 at the nuclear pore complex. Mol Cell Biol.

[46] Dahan-Pasternak N, Nasereddin A, Kolevzon N, Pe’er M, Wong W, Shinder V (2013). PfSec13 is an unusual chromatin-associated nucleoporin of *Plasmodium falciparum* that is essential for parasite proliferation in human erythrocytes. J Cell Sci.

[47] Madeira L, DeMarco R, Gazarini ML, Verjovski-Almeida S, Garcia CR (2003). Human malaria parasites display a receptor for activated C kinase ortholog. Biochem Biophys Res Commun.

[48] Buensuceso C, Woodside D (2001). The WD protein Rack1 mediates protein kinase C and integrin-dependent cell migration. J Cell Sci.

[49] Chen S, Spiegelberg BD, Lin F, Dell EJ, Hamm HE (2004). Interaction of Gbetagamma with RACK1 and other WD40 repeat proteins. J Mol Cell Cardiol.

[50] Sartorello R, Amaya MJ, Nathanson MH, Garcia CRS (2009). The Plasmodium receptor for activated C kinase protein inhibits Ca(2+) signaling in mammalian cells. Biochem Biophys Res Commun.

