# Supplementary material for: A WD40-repeat protein unique to malaria parasites associates with adhesion protein complexes and is crucial for blood stage progeny
Source: Malar J. 2015 Nov 4;14:435. doi: 10.1186/s12936-015-0967-x (PMC4634918; doi:10.1186/s12936-015-0967-x)
Supplement: Supplementary file 1 — 10.1186/s12936-015-0967-x Purity of the recombinant PfWLP1 proteins and reactivity of the respective antisera. [file 12936_2015_967_MOESM1_ESM.pdf]

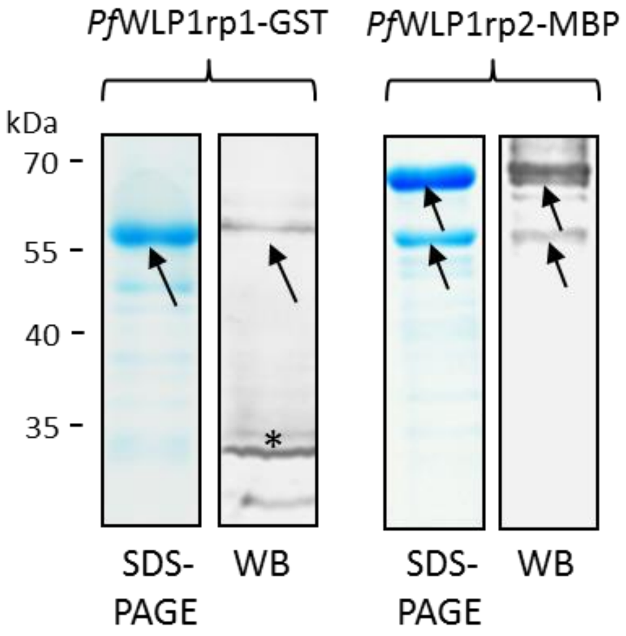


**Additional file 1 Purity of the recombinant *Pf*WLP1 proteins and reactivity of the respective antisera**

Affinity-purified recombinant *Pf*WLP1rp1-GST and *Pf*WLP1rp2-MBP were subjected to SDS-PAGE followed by Coomassie blue staining or Western blotting (WB), using the respective mouse antisera. Arrows indicate recombinant *Pf*WLP1rp1-GST (58 kDa) and *Pf*WLP1rp2-MBP (65 kDa), the lower protein band represents a shorter protein due to incomplete expression. The asterisk indicates the GST protein.
