# Supplementary material for: A WD40-repeat protein unique to malaria parasites associates with adhesion protein complexes and is crucial for blood stage progeny
Source: Malar J. 2015 Nov 4;14:435. doi: 10.1186/s12936-015-0967-x (PMC4634918; doi:10.1186/s12936-015-0967-x)
Supplement: Supplementary file 2 — 10.1186/s12936-015-0967-x Homology analyses of PWLP1 proteins. [file 12936_2015_967_MOESM2_ESM.pdf]

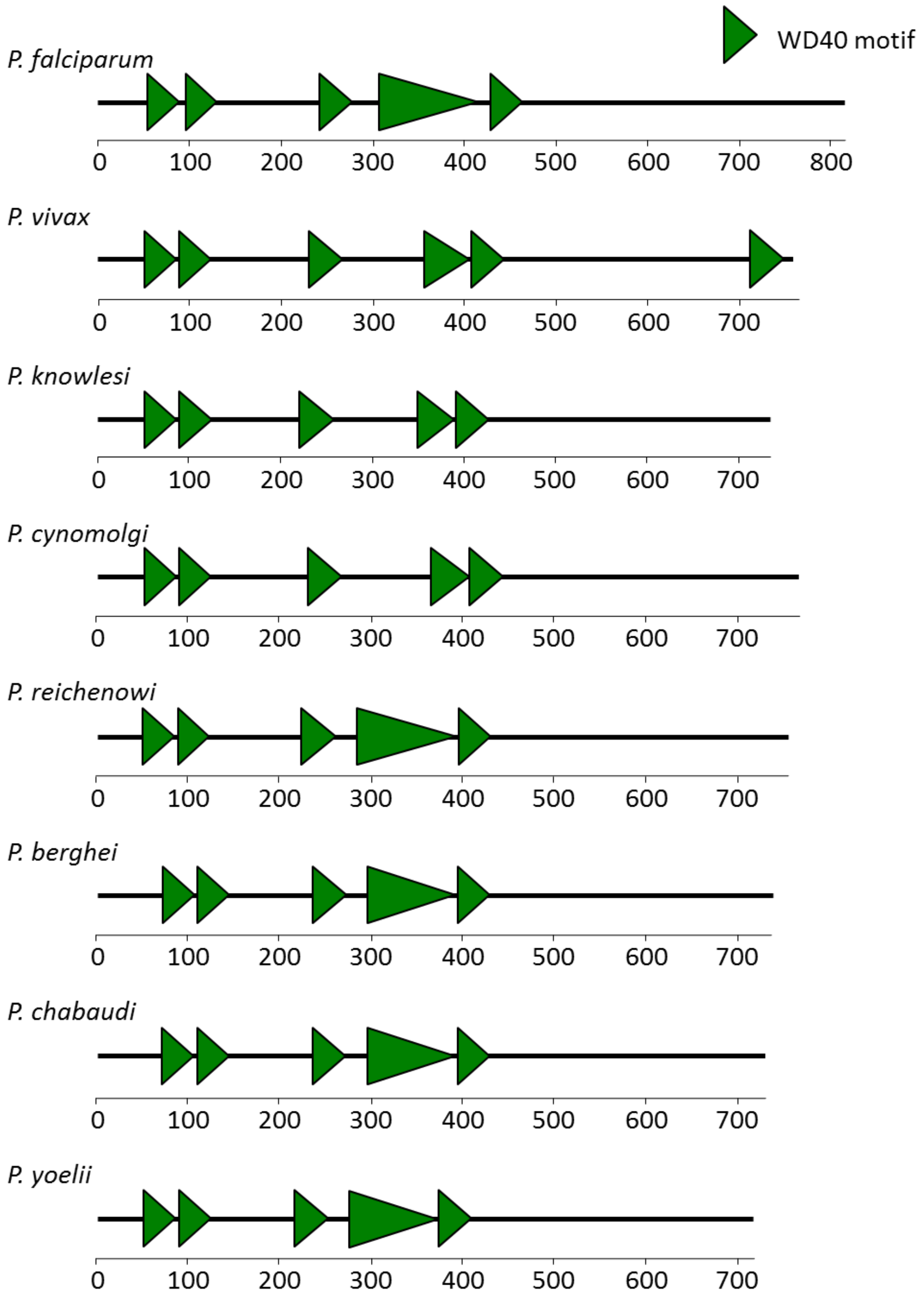

Additional file 2 Homology analyses of *P*WLP1 proteins

Comparative schematic of plasmodial WLP1 proteins. The annotated WD40 motifs (green triangles) are represented.
