# Supplementary material for: A WD40-repeat protein unique to malaria parasites associates with adhesion protein complexes and is crucial for blood stage progeny
Source: Malar J. 2015 Nov 4;14:435. doi: 10.1186/s12936-015-0967-x (PMC4634918; doi:10.1186/s12936-015-0967-x)
Supplement: Supplementary file 3 — 10.1186/s12936-015-0967-x RT-PCR genomic DNA control and PfWLP1 relocalization in maturing schizonts. [file 12936_2015_967_MOESM3_ESM.pdf]

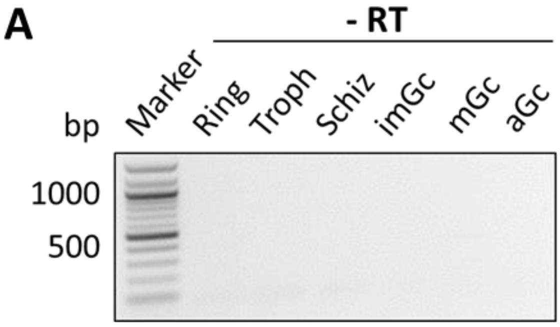

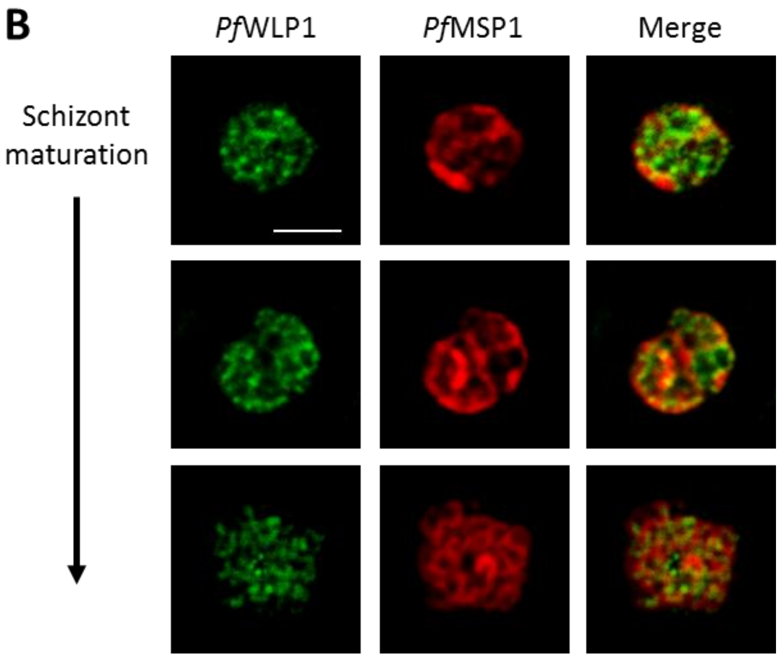

Additional file 3 RT-PCR genomic DNA control and *Pf*WLP1 relocalization in maturing schizonts

A. RT-PCR genomic DNA control. Mock-treated RNA samples lacking reverse transcriptase (-RT) in combination with *pfaldolase* primers were used to confirm that genomic DNA was absent in all cDNA samples used for diagnostic RT-PCR as shown in Figure 1C.

B. Relocalization of *Pf*WLP1 in maturing schizonts. *Pf*WLP1 was immunolabelled with anti-*Pf*WLP1-rp2 antisera (green); the schizont plasma membrane was visualized with antisera against *Pf*MSP1 (red).Bar, 5 µm. Data are representative of three independent experiments.
