# Supplementary material for: A WD40-repeat protein unique to malaria parasites associates with adhesion protein complexes and is crucial for blood stage progeny
Source: Malar J. 2015 Nov 4;14:435. doi: 10.1186/s12936-015-0967-x (PMC4634918; doi:10.1186/s12936-015-0967-x)
Supplement: Supplementary file 4 — 10.1186/s12936-015-0967-x IFA negative and PfWLP1rp1 antibody controls. [file 12936_2015_967_MOESM4_ESM.pdf]

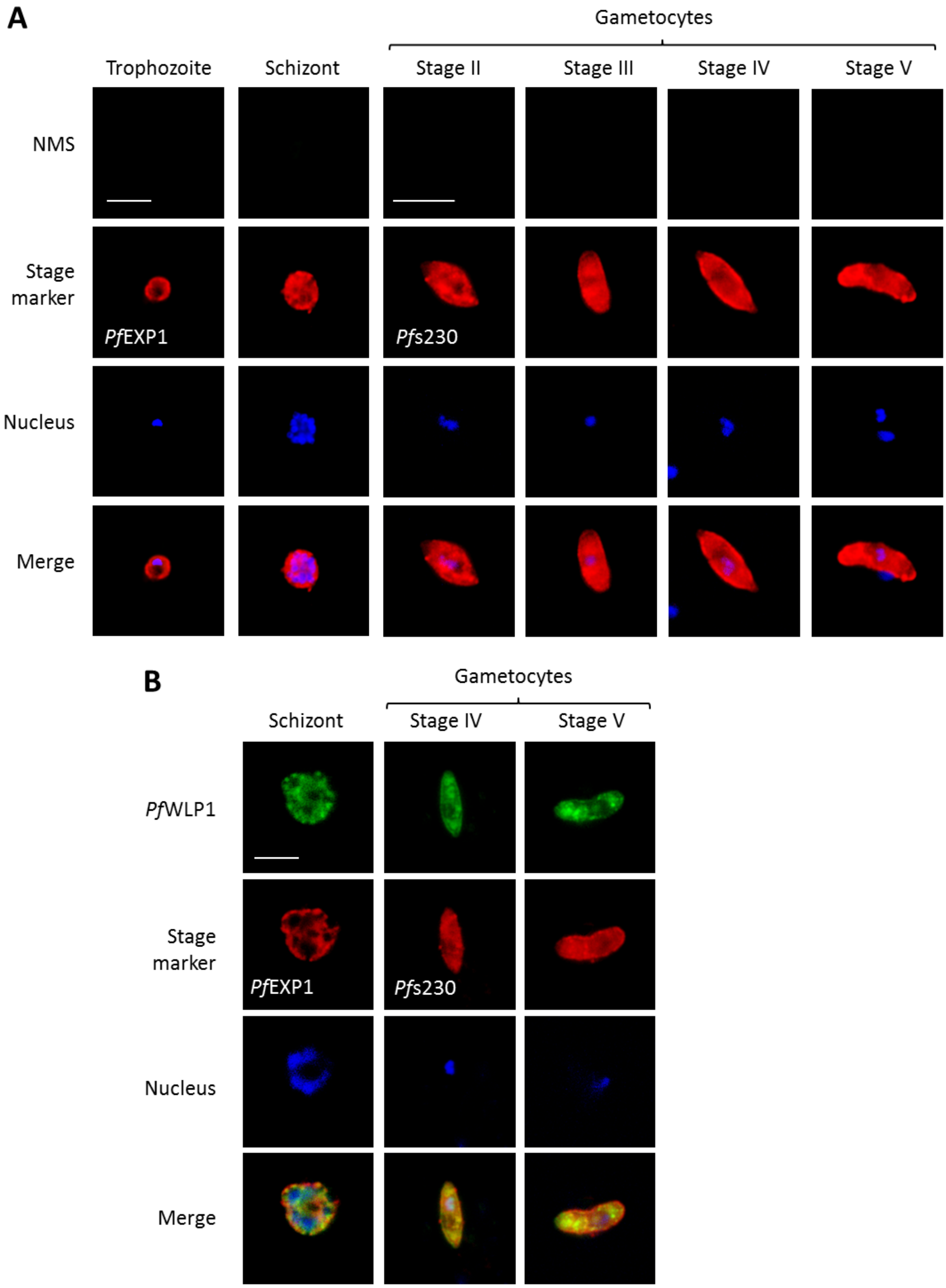
A
Gametocytes
Trophozoite
Schizont
Stage II
Stage III
Stage IV
Stage V
NMS
Stage marker
PfEXP1
Pfs230
Nucleus
Merge
B
Gametocytes
Schizont
Stage IV
Stage V
PfWLP1
Stage marker
PfEXP1
Pfs230
Nucleus
Merge

Additional file 4 IFA negative and *Pf*WLP1rp1 antibody controls

A. IFA negative control. Sera of non-immunized mice (NMS) for immunolabelling of mixed blood stages (green).

B. *Pf*WLP1rp1 antibody control. Mixed blood stages were immunolabelled with anti-*Pf*WLP1rp1 antisera (green). The asexual blood stages and gametocytes (stages II-V) were visualized by rabbit anti-EXP-1 and anti-*Pf*s230 antisera, respectively (red). The parasite nuclei were highlighted by Hoechst stain (in blue). Bar, 5 µm. Data are representative of two independent experiments.
