# Supplementary material for: A WD40-repeat protein unique to malaria parasites associates with adhesion protein complexes and is crucial for blood stage progeny
Source: Malar J. 2015 Nov 4;14:435. doi: 10.1186/s12936-015-0967-x (PMC4634918; doi:10.1186/s12936-015-0967-x)
Supplement: Supplementary file 5 — 10.1186/s12936-015-0967-x Co-labelling of PfWLP1 with alpha-tubulin. [file 12936_2015_967_MOESM5_ESM.pdf]

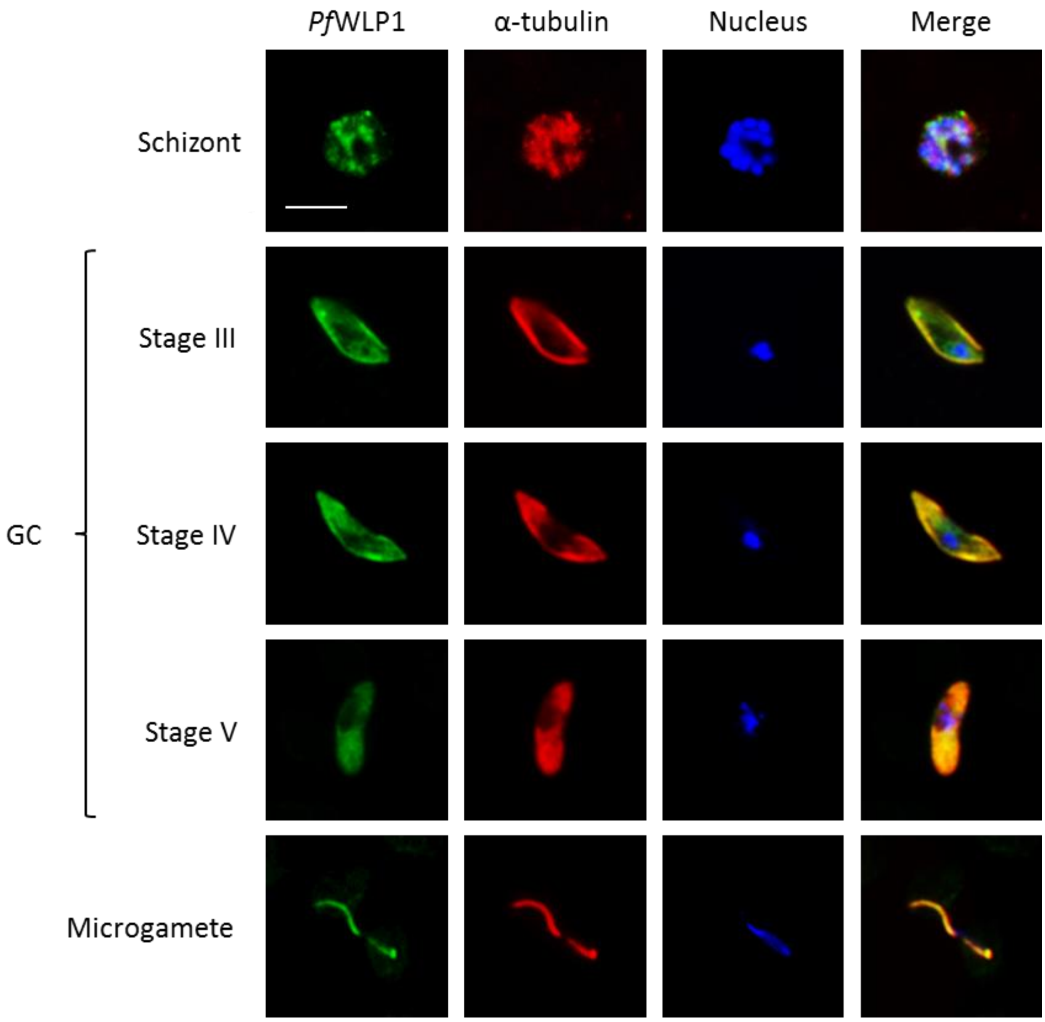

Additional file 5 Co-labelling of *Pf*WLP1 with alpha-tubulin

Mixed blood stages and activated gametocytes were immunolabelled with anti-*Pf*WLP1rp2 antisera (green); the microtubuli were visualized with antisera against alpha-tubulin (red). The parasite nuclei were highlighted by Hoechst nuclear stain (in blue). Bar, 5 µm.
