# Supplementary material for: A WD40-repeat protein unique to malaria parasites associates with adhesion protein complexes and is crucial for blood stage progeny
Source: Malar J. 2015 Nov 4;14:435. doi: 10.1186/s12936-015-0967-x (PMC4634918; doi:10.1186/s12936-015-0967-x)
Supplement: Supplementary file 6 — 10.1186/s12936-015-0967-x Expression of PfWLP1 in WT and knock-out gametocytes. [file 12936_2015_967_MOESM6_ESM.pdf]

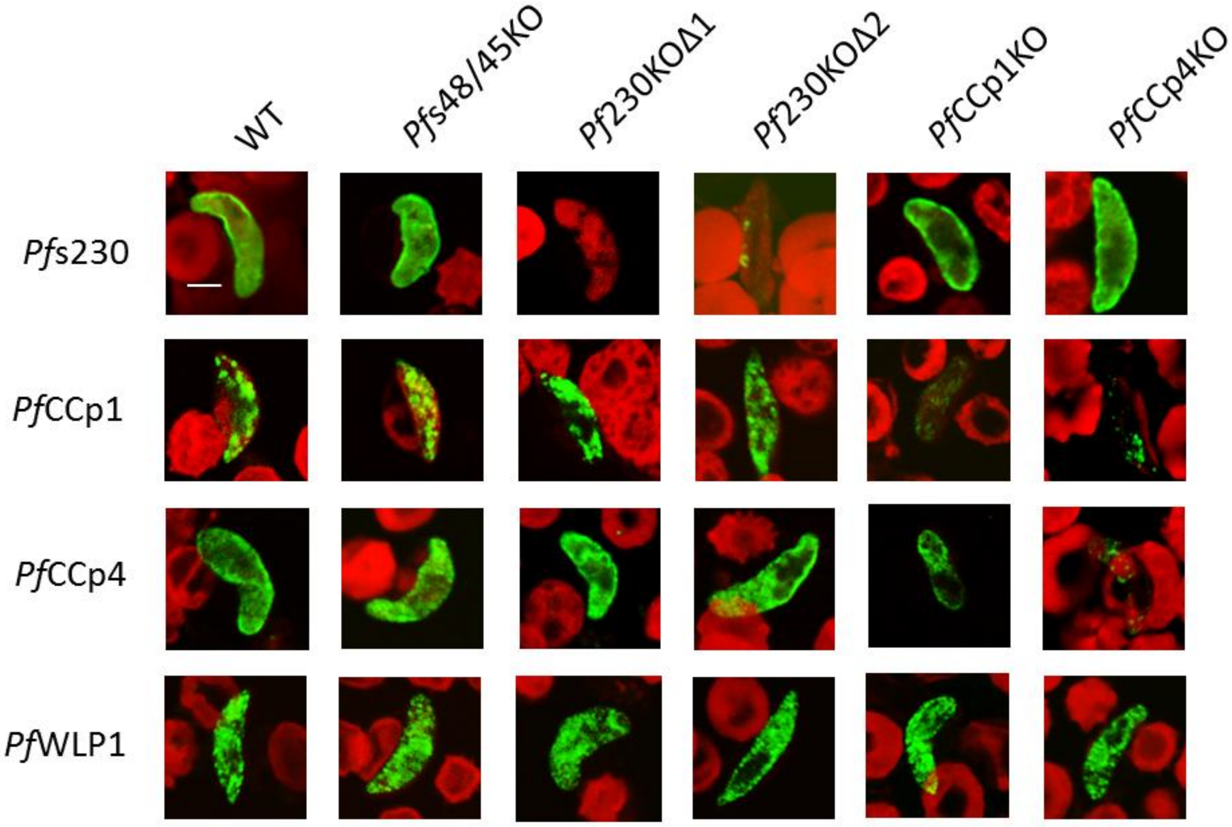

Additional file 6 Expression of *Pf*WLP1 in WT and knock-out gametocytes

Non-activated gametocytes of WT strain NF54 and of gene knock-out lines *Pf*s230-delta1, *Pf*s230-delta2, *Pf*s48/45KO, *Pf*CCp1KO, and *Pf*CCp4KO were immunolabelled with antisera directed against *Pf*s230, *Pf*CCp1 and *Pf*CCp4 of *Pf*WLP1 (green) in these lines. Erythrocytes and gametocytes were counterlabelled with Evans Blue (red). Bar, 2 µm.
