# Supplementary material for: A WD40-repeat protein unique to malaria parasites associates with adhesion protein complexes and is crucial for blood stage progeny
Source: Malar J. 2015 Nov 4;14:435. doi: 10.1186/s12936-015-0967-x (PMC4634918; doi:10.1186/s12936-015-0967-x)
Supplement: Supplementary file 7 — 10.1186/s12936-015-0967-x Strategy for pfwlp1 gene locus disruption and generation of a HA-tagged PfWLP1 parasite line. [file 12936_2015_967_MOESM7_ESM.pdf]

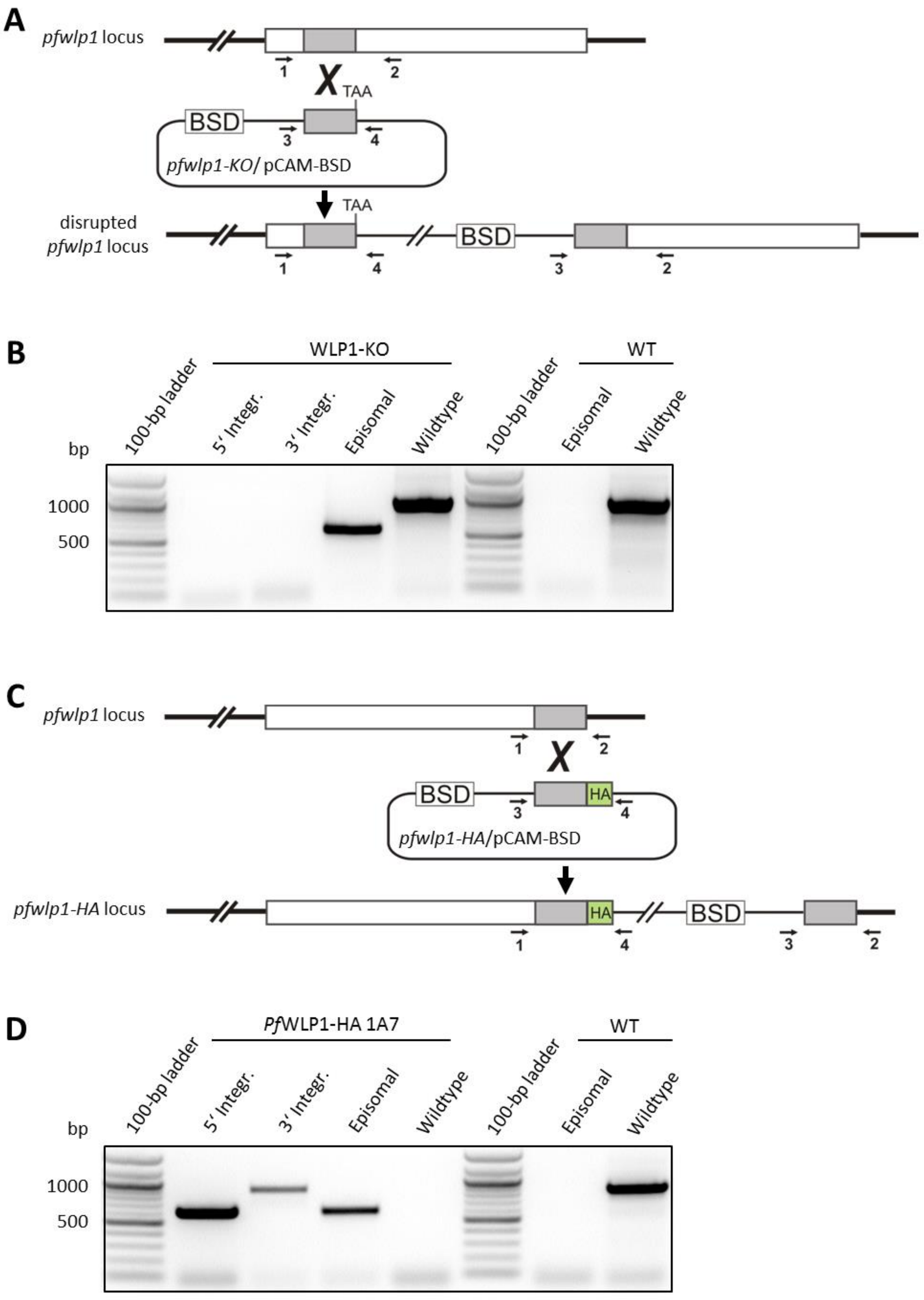
A
pfwlp1 locus
1
2
X
TAA
BSD
3
4
pfwlp1-KO/ pCAM-BSD
TAA
disrupted
pfwlp1 locus
1
4
BSD
3
2
B
WLP1-KO
WT
100-bp ladder
5′ Integr.
3′ Integr.
Episomal
Wildtype
100-bp ladder
Episomal
Wildtype
bp
1000
500
C
pfwlp1 locus
1
2
X
BSD
3
HA
4
pfwlp1-HA/pCAM-BSD
pfwlp1-HA locus
HA
BSD
1
4
3
2
D
PfWLP1-HA 1A7
WT
100-bp ladder
5′ Integr.
3′ Integr.
Episomal
Wildtype
100-bp ladder
Episomal
Wildtype
bp
1000
500

Additional file 7 Strategy for *pfwlp1* gene locus disruption and generation of a HA-tagged *Pf*WLP1 parasite line

A. Schematic of the single-crossover homologous integration strategy to generate a *pfwlp1* gene-disruptant mutant. Arrows with numbers mark the position of primers used to investigate the integration of disruption vector *pfwlp1*-KO pCAM-BSD.

B. Diagnostic PCR on isolated DNA was performed to investigate gene locus integration of the *pfwlp1-KO* pCAM-BSD vector (WLP1-KO) and no vector integration was detected after 20 weeks of cultivation. Primers 1 and 4 were used for amplification of 5´-integration and primers 2 and 3 for 3´-integration of the vector. Episomal DNA was amplified by primers 3 and 4 and WT *pfwlp1* locus DNA by primers 1 and 2. DNA from mock control (WT) was used for negative control.

C. Schematic of the single-crossover homologous integration strategy to generate a *pfwlp1* HA-tagged mutant. Arrows with numbers mark the position of primers used to investigate the integration of the *pfwlp1*-HA pCAM-BSD vector.

D. Diagnostic PCR on isolated DNA was performed to confirm gene locus integration of the *pfwlp1-HA* pCAM-BSD vector in NF54 clone 1A7 (WLP1-HA 1A7). Primers 1 and 4 were used for amplification of the 5´-integration and primers 3 and 2 for 3´-integration of the vector. Episomal DNA was amplified by primers 3 and 4 and WT *pfwlp1* locus DNA by primers 1 and 2. DNA from mock control (WT) was used for negative control.
