# Supplementary material for: A WD40-repeat protein unique to malaria parasites associates with adhesion protein complexes and is crucial for blood stage progeny
Source: Malar J. 2015 Nov 4;14:435. doi: 10.1186/s12936-015-0967-x (PMC4634918; doi:10.1186/s12936-015-0967-x)
Supplement: Supplementary file 8 — 10.1186/s12936-015-0967-x Co-detection of PfWLP1-HA using anti-HA and anti-PfWLP1 antibodies. [file 12936_2015_967_MOESM8_ESM.pdf]

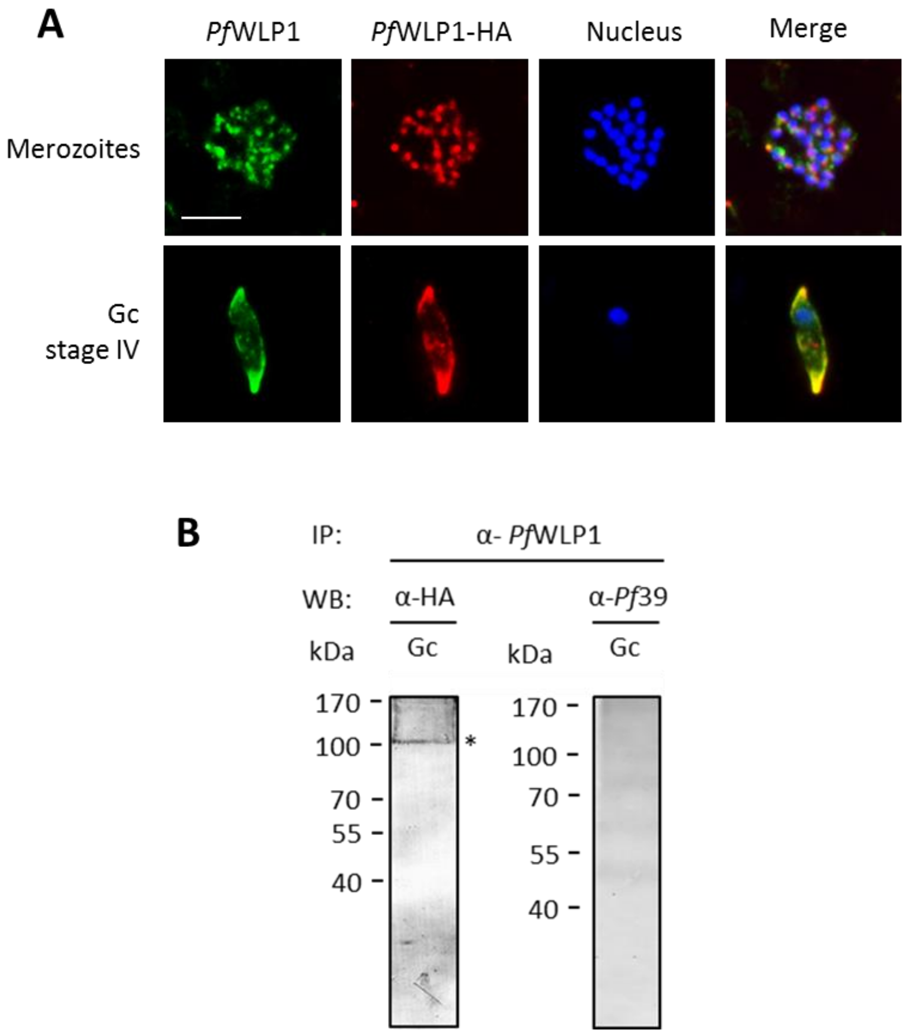


**Additional file 8 Co-detection of *Pf*WLP1-HA using anti-HA and anti-*Pf*WLP1 antibodies**

A. Labelling of *Pf*WLP1-HA with anti-*Pf*WLP1 antisera. Mixed blood stages of line *Pf*WLP1-HA were immunolabelled with both anti-*Pf*WLP1rp2 (green) and anti-HA (red) antisera. The parasite nuclei were highlighted by Hoechst nuclear stain (in blue). Bar, 5 µm.

B. Immunoprecipitation of *Pf*WLP1-HA using anti-*Pf*WLP1 antisera. Lysates of enriched schizonts of line *Pf*WLP1-HA were immunoprecipitated with anti-*Pf*WLP1rp2 antisera, followed by Western blot analysis using anti-HA antibody to detect *Pf*WLP1-HA (~108 kDa). Data are representative of two to three independent experiments.
